# Advanced Treatment of Landfill Leachate Induced Dissolved Organic Nitrogen (DON) and Its Influence on the Estuarine Algal Community

**DOI:** 10.1021/acsestwater.4c01265

**Published:** 2025-08-19

**Authors:** Harsh V. Patel, Md Redowan Rashid, Md Ashik Ahmed, Lifeng Zhang, Brian Brazil, Wenzheng Yu, Hans W. Paerl, Renzun Zhao

**Affiliations:** † Department of Civil, Architectural, and Environmental Engineering, 3616 North Carolina A&T State University, Greensboro, North Carolina 27411, United States; ‡ Joint School of Nanoscience and Nanoengineering, 3616 North Carolina A&T State University, Greensboro, North Carolina 27401, United States; § 49244 Waste Management, Inc., Gaithersburg, Maryland 20878, United States; ∥ State Key Laboratory of Environmental Aquatic Chemistry, Research Center for Eco-Environmental Sciences, Chinese Academy of Sciences, Beijing 100084, China; ⊥ Institute of Marine Sciences, University of North Carolina at Chapel Hill, Morehead City, North Carolina 28557, United States

**Keywords:** landfill leachate, dissolved organic nitrogen (DON), fenton treatment, granular activated carbon (GAC), in situ algal bioassays

## Abstract

Landfill leachate is a major source of refractory dissolved organic nitrogen (rDON), which can exacerbate eutrophication and harmful algal blooms in downstream aquatic ecosystems. This study evaluates the effectiveness of two advanced physicochemical treatments—Fenton oxidation and granular activated carbon (GAC) adsorption—for rDON removal from biologically treated landfill leachate blended with sewage, and their impacts on the estuarine algal (phytoplankton) community with *in situ* algal bioassays. Fenton oxidation achieved 52%–60% rDON removal by converting rDON into ammonium nitrogen (NH_4_^+^-N), enhancing its biodegradability and suitability for subsequent biological treatments. In contrast, GAC adsorption achieved higher removal efficiencies (86%–92%) by physically adsorbing nitrogenous species, including rDON and NH_4_^+^-N, without altering their chemical structure. We deployed *in situ* algal bioassays to analyze the impacts of advanced wastewater treatment processes on the algal growth dynamics. Bioassays revealed distinct effects on algal growth: Fenton treatment temporarily increased algal biomass due to elevated NH_4_^+^-N levels, while GAC treatment mitigated nutrient availability, inhibiting algal proliferation. While GAC was more effective overall, its regeneration requirements and associated costs pose applicability challenges. Fenton treatment is best suited as a pretreatment step to enhance rDON biodegradability.

## Graphical abstract

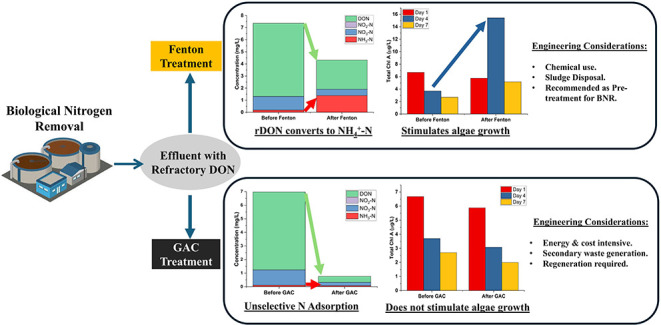

## 1 Introduction

Landfill leachate is a complex wastewater source characterized by elevated concentrations of organic matter, ammonia/ammonium, heavy metals, and recalcitrant pollutants, including dissolved organic nitrogen (DON).^1,2^ DON comprises a heterogeneous mixture of organic nitrogen-containing compounds that are not readily removed through conventional filtration or biological treatment processes.^3^ Although historically overshadowed by concerns over ammonia/ammonium (NH_*x*_) and nitrate, DON has emerged as a significant contributor to nitrogen loading in aquatic environments.^4^ Numerous studies have shown that DON can serve as a bioavailable nitrogen source, promoting harmful algal blooms (HABs), especially in nitrogen-limited freshwater and estuarine systems.^5–10^ Recently, there has been an increase in the occurrence of toxic cyanobacterial blooms (CyanoHABs) in the rivers and estuaries of North Carolina and worldwide.^11^ Although the loading of inorganic nitrogen into these waterways has decreased over time, long-term data reveal that organic nitrogen loading has risen, coinciding with an increase in harmful algal blooms.^12^ Studies have shown that DON, including proteinaceous and humic substance-derived forms, plays a crucial role in stimulating the growth of HAB taxa such as cyanobacteria and dinoflagellates.^13^ Given DON’s ecological and public health risks, particularly from sources such as landfill leachate, it is essential to develop effective treatment strategies to mitigate these environmental impacts. Current wastewater treatment technologies, including nitrification/denitrification, primarily target inorganic nitrogen, and are insufficient for removing organic nitrogen compounds.

The cotreatment of landfill leachate with municipal sewage has become a common practice to dilute high-strength leachate and leverage existing wastewater infrastructure.^14–18^ However, the integration of these streams introduces treatment challenges due to the refractory nature of leachate-derived organic nitrogen. The Modified Ludzack-Ettinger (MLE) process, a widely implemented biological nutrient removal configuration, facilitates nitrification and denitrification and has been employed to manage such blended wastewater. Yet, despite its effectiveness in transforming inorganic nitrogen species, the MLE process often leaves a substantial fraction of DON untreated, particularly under imbalanced carbon-to-nitrogen ratios or when complex organic matter is present.^19–25^

Due to incomplete removal of DON via biological treatment, discharged effluents with recalcitrant DON can potentially stimulate algal growth in natural water bodies. To overcome the limitations of biological treatment alone, advanced post-treatment technologies such as Fenton oxidation and granular activated carbon (GAC) are commonly applied. The Fenton process generates hydroxyl radicals that aggressively oxidize recalcitrant organic compounds, potentially breaking down DON precursors into more bioavailable intermediates. Despite its effectiveness, Fenton treatment has only been evaluated for overall organic matter removal, rather than its efficacy in removing untreated DON postbiological treatment.^26–30^ GAC provides an adsorption-based mechanism to remove residual organic nitrogen and low-molecular-weight byproducts. Like the Fenton treatment, no previous studies have addressed the efficacy of GAC treatment for untreated DON postbiological treatment.^31,32^ While these technologies have demonstrated enhanced removal of persistent organics, the specific transformation pathways and fate of DON during such multistage treatment remain poorly understood.

This study is one of the first to apply advanced physicochemical post-treatment (Fenton oxidation and GAC) to landfill leachate–sewage cotreated effluents while directly evaluating their impacts on estuarine algal community dynamics using in situ bioassays. Unlike previous fractionation-based or monoculture-based assessments, our approach captures realistic ecosystem responses to advanced treatment strategies. Additionally, the study evaluates the potential ecological risks posed by the final effluent *using in situ* algal bioassays to assess stimulation of algal growth. The specific objectives of this research are to (i) quantify DON removal at each stage of treatment, (ii) characterize chemical alterations in DON composition, and (iii) determine the effect of Fenton and GAC-treated effluent on algal bloom potential. Findings from this work will inform the development of integrated leachate management strategies that effectively minimize both pollutant discharge and downstream negative ecological impacts (eutrophication and HABs).

## 2 Methods and Materials

### 2.1 Biological Treatment of Blended Landfill Leachate and Sewage

For all the experiments in this study, all physicochemical treatments (Fenton oxidation and GAC adsorption) were applied to the effluents from a biological nutrient removal (BNR) process conducted in sequencing batch reactors (SBR) fed with a blend of landfill leachate and sewage. The landfill leachate sample was collected from municipal solid waste landfills in Virginia and Arkansas, USA, and the sewage sample was collected from a local wastewater treatment plant in North Carolina, USA. Two SBRs, labeled R1 and R2, were operated in this study (Figure S13). The characteristics of raw sewage and raw landfill leachate samples fed to reactor R1 and R2 are shown in Tables S1 and S2. Both reactors were made from poly­(methyl methacrylate) (PMMA) with a total volume of 5 L and a working volume of 3 L. Based on the literature review, leachate was mixed with municipal wastewater for cotreatment using a 0.02% to 10% volumetric ratio, from which studies suggest that using a less than 1% volumetric ratio had decreased effect on any treatment processes. The volumetric ratio was also found to depend on leachate age and characteristics.^33–40^ Hence, to determine the leachate loading each reactor could accept without impairing nitrification, we first ran both reactors on municipal wastewater alone until steadystate nitrification was established. Leachate was then introduced gradually, starting at 0.2% (v/v) and increasing in equal increments every four to five cycles. Reactor 1, which received the highCOD leachate, showed clear nitrification inhibition once the leachate fraction exceeded 0.8%. In contrast, Reactor 2, dosed with the lowCOD leachate, maintained complete nitrification up to 3% leachate. These threshold concentrations reflect the differing organic loads of the two leachates and define the maximum blending ratios that still allowed full nitrification. The stepwiseloading approach mimics realistic cotreatment practice in wastewater treatment plants and underscores the need to consider leachate strength when setting operational limits. The percentage nitrification was calculated for each landfill leachate and sewage ratio to determine the optimum ratio as shown in Figure S1.

A 24hour hydraulic retention time (HRT) was originally adopted following Hu et al.,^41^ implemented as three 8 hour cycles per day—each comprising 4 hours aerobic, 3 hours anoxic, and 1 hour for settling, decanting, feeding, and idle—but this regime failed to deliver adequate nitrification, denitrification, or BOD removal despite external carbon addition. Performance improved after switching to two 12 hour cycles per HRT (7.5 hours aerobic, 3 hours anoxic, 0.5 hour postaerobic, and 1 hour for settling and ancillary steps), yet denitrification and BOD removal were still suboptimal. A subsequent revision to 7 hours aerobic, 3.5 hours anoxic, 0.5 hour postaerobic, and 1 hour settling enhanced denitrification but left BOD removal incomplete. The final configuration—6.5 hours aerobic, 3.5 hours anoxic, 1 hour postaerobic, and 1 hour for settling/decanting/idle—achieved complete nitrification and denitrification together with substantial BOD reduction and was therefore adopted for ongoing operation. This information is also shown in Table S3. The solids retention time (SRT) was maintained at 15 days for both reactors.

Additional operational conditions included a mixing speed of 200 ± 6 rpm and a temperature of 20 ± 1 °C for both reactors. The mixed liquor suspended solids (MLSS) concentration was approximately 4,000 mg/L in both R1 and R2. Airflow rates differed slightly, with R1 set at 0.8 L/min and R2 at 0.5 L/min. Dissolved oxygen (DO) levels in both reactors ranged from 1 to 2 mg/L, and methanol was added as a carbon source at concentrations of 0.77 L/m^3^ for R1 and 0.87 L/m^3^ for R2 to support denitrification. These conditions were carefully controlled to optimize effective biological nitrogen removal in both SBRs. The influent and effluent characteristics of R1 and R2 is shown in Table S4.

### 2.2 Physiochemical Treatment

#### 2.2.1 Fenton Treatment

The experiments were conducted in batch reactors using 50 mL beakers (Corning, Corning, New York, USA), each containing a sample volume of 30 mL. The Fenton process is executed in four distinct phases: pH adjustment, oxidation, neutralization, and sedimentation. Initially, the pH of the samples was adjusted to 3.1 ± 0.04 using sulfuric acid (VWR, Radnor, Pennsylvania, USA) and sodium hydroxide (VWR, Radnor, Pennsylvania, USA) to achieve optimum conditions for the Fenton reaction. Following this adjustment, iron­(II) sulfate heptahydrate (VWR, Radnor, Pennsylvania, USA) was added in appropriate proportions as part of the Fenton reagent, and the mixture was agitated for 15 min using a magnetic stirrer. Subsequently, hydrogen peroxide (H_2_O_2_, 30% w/w) (VWR, Radnor, Pennsylvania, USA) was introduced and the mixture was stirred at 110 rpm for 1 min to initiate the Fenton reaction at room temperature (25 °C). The stirring speed was reduced to 30 rpm, allowing the reaction to proceed for 40 min.

After the oxidation phase, the pH was adjusted between 8 and 9 using sodium hydroxide to facilitate the decantation of iron hydroxide and the decomposition of any remaining hydrogen peroxide. The mixture was allowed to settle for 1 h, after which the supernatants were collected and filtered through a 0.45 μm porosity filter paper (Whatman, Wilmington, Delaware, USA). Filtered samples were stored at 4 °C until further analysis. Multiple trials utilizing the optimum dosage were conducted to determine the optimum reaction time, with each batch comprising four beakers. The total testing duration was 40 min, with individual reaction times varying from 5 to 40 min, leading to the identification of optimum conditions. The results have been described in the Supporting Information along with Figures S2 and S3.

#### 2.2.2 GAC Treatment

The dosage range for Granular Activated Carbon (GAC) (Strem Chemicals, Inc., Newburyport, Massachusetts, USA) was initially determined based on the relevant literature^42–44^ and was tailored to the chemical oxygen demand (COD) levels of our samples. The characteristics of the GAC has been provided in Table S6. The established range for GAC was 5–25 g/L. For the experiments, a sample volume of 30 mL was placed in a 50 mL conical flask, to which varying amounts of GAC were added. The flasks were then placed on an orbital shaker and agitated continuously at 25 °C and 200 rpm for 24 h. Following agitation, the treated samples were allowed to settle for 30 min. The supernatant was then filtered using 0.45 μm porosity filter paper (Whatman, Wilmington, Delaware, USA), and the filtered samples were stored at 4 °C for subsequent testing.

Multiple experiments were conducted to determine the optimum contact time using GAC dosage. Twelve conical flasks were used in the batch process. The testing duration was set to 24 h, and samples were extracted every 2 h for further analysis. This allowed the determination of the optimum contact time. The results have been described in the Supporting Information along with Figures S4 and S5. To evaluate the adsorption capacity, kinetics, and isotherm parameters, the following data were recorded: concentration of dissolved organic nitrogen (DON) over time (C_t_), equilibrium time (t_e_ in hours), initial DON concentration (C_0_), equilibrium DON concentration (C_e_), and adsorbent dose. These measurements enabled the calculation of the adsorption capacity (q_m_ in mg/g) and isotherm coefficients. Linear forms of the Langmuir and Freundlich isotherms were fitted for the GAC adsorption data, as represented by eqs 1 and 2.

1 $$\frac{C_{e}}{Q_{e}}=\frac{1}{q_{max}b}+\frac{C_{e}}{q_{max}}$$

2 $$\log Q_{e}=\log K_{F}+\left(\frac{1}{n}\right)\log C_{e}$$

### 2.3 Chemical Analysis and Characterization

#### 2.3.1 Water Quality Analysis

The following chemical parameters for filtered water samples were evaluated in our experiments: chemical oxygen demand (COD), total nitrogen (TN), ammonium nitrogen (NH_4_^+^-N), nitrate nitrogen (NO_3_^–^-N), nitrite nitrogen (NO_2_^–^-N), and dissolved organic nitrogen (DON). The DON concentration was calculated by subtracting the concentrations of nitrate, nitrite, and ammonium nitrogen from the total nitrogen content, as defined by the equation:^2^

$$\mathrm{DON}=\mathrm{TN}-{\mathrm{NH}}_{4}^{+}\mathrm{-N}-{\mathrm{NO}}_{3}^{-}\mathrm{-N}-{\mathrm{NO}}_{2}^{-}\mathrm{-N}$$

The total nitrogen concentration in the samples was determined using the persulfate digestion method with a Hach TNT 826 test kit (Hach, Loveland, Colorado, USA). Nitrate levels were quantified using the dimethylphenol technique, while nitrite concentrations were assessed through the diazotization method, both of which were also performed using Hach TNT 835 and 839 test kits (Hach, Loveland, Colorado, USA). Ammonium nitrogen concentrations in the wastewater samples were measured using the salicylate method with a Hach TNT 830 test kit (Hach, Loveland, Colorado, USA). COD was measured using the Reactor Digestion method with a Hach TNT 825 test kit (Hach, Loveland, Colorado, USA). Each analytical procedure was replicated three times to ensure the statistical validity of the results.

#### 2.3.2 Dissolved Organic Matter Spectral Characterization

Fourier transform infrared (FTIR) spectra of the freeze-dried powdered samples were recorded using an Agilent 670 FTIR Spectrometer equipped with an Attenuated Total Reflectance (ATR) accessory (Agilent, Santa Clara, California, USA). Each sample was placed in contact with a diamond ATR crystal, and a single spectrum was acquired from each sample across a spectral range of 4000–550 cm^–1^. Measurements were performed in the transmission mode with a resolution of 8 cm^–1^, averaging 64 scans per spectrum. The freeze-drying process was carried out using a Labconco FreeZone 2.5 L Freeze-Dryer System (Labconco, Kansas City, Missouri, USA). Prior to freeze-drying, samples were first stored at – 20 °C to ensure complete freezing. Subsequently, samples (200 mL in 300 mL bottles) were freeze-dried using a Labconco FreeZone 2.5 L Freeze-Dryer under vacuum conditions of 0.03–0.05 mbar at – 50 °C for 36–48 hours until a consistent dry powder was obtained. This procedure was chosen to concentrate dissolved organic matter while preserving functional group structures for FTIR and NMR analyses. The results and discussion for FTIR analysis are presented in the Supporting Information with Figures 4 and S10.

The ^13^C nuclear magnetic resonance (NMR) spectra of the freeze-dried samples were recorded using a 400 MHz Agilent 400 NMR instrument (Agilent, Santa Clara, California, USA). For the analysis, 50 mg of each sample was dissolved in 1 mL deuterium oxide (Thermo Fisher Scientific, Waltham, Massachusetts, USA). To adequately capture all chemical shifts, each spectrum was acquired over a period of 24 to 36 h owing to the inherently low sensitivity of ^13^C. Water suppression techniques were employed to reduce the intensity of the water peak at a frequency of 4.66 ppm (ppm), which allowed for better resolution of peaks with lower intensities. Each spectrum acquisition comprised 115,000 scans, utilizing a 1-s acquisition time, a 1-s recycle delay, and a pulse angle of 45 °. Before analysis, each spectrum was processed, including a 30 Hz line broadening and baseline correction to enhance the quality of the data. The result and discussion of NMR spectral analysis are presented in the Supporting Information with Figures 4 and S11.

### 2.4 Algal Bioassay and HPLC Analysis of Community Composition

The treated wastewater effluents were sent to the Institute of Marine Sciences at the University of North Carolina at Chapel Hill for *in situ* algal bioassays. Bioassays were used to evaluate the nutritional impacts of the treated samples on the growth of the planktonic algal community (phytoplankton) containing representative algal groups, including harmful algal bloom taxa, in the Neuse River Estuary (NRE). The experiments were conducted over a period of 7 days.

Water from Site 100 of the ModMon water quality monitoring project (https://paerllab.web.unc.edu/modmon/) in the Neuse River Estuary (NRE) was used to assess the effects of treatment vs control samples. The NRE serves as an effective model for studying local algal dynamics because of the observed increases in harmful cyanobacterial and dinoflagellate blooms that have been linked to nitrogen enrichment.^45,46^ The experiments were conducted in 4 L polyethylene, 80% transparent to photosynthetically active radiation (PAR) Cubitainers (Hedwin Corp., Baltimore, MD, USA), with triplicate samples for untreated controls and respective treatments. The Cubitainers were cleaned by rinsing with 0.01 M HCl, followed by D.I. and NRE sample water used for the bioassay. NRE water was dispensed in a polyethylene tank that was constantly agitated to ensure homogeneity of water added to Cubitainers. All Cubitainers were then supplemented with 10 mgC/L sodium bicarbonate to avoid inorganic C limitation during the incubation period. Effluents collected from the Fenton treatment and GAC treatment were added to the Cubitainers to make the final total nitrogen approximately 30 μM·N. The Cubitainers were then placed in a corral in an outdoor pond adjacent to the Institute of Marine Sciences for 7 days. Corrals were covered by a layer of neutral density screening, which reduced incident radiation by ∼30% to avoid photoinhibition during algal growth. Daily subsamples from the Cubitainers (T1–T7) were collected for nutrient analysis on Whatman GF/F (0.7 μm porosity) filtrate. Filter retentate containing particulate matter were then used for measuring algal growth through chlorophyll a/b analysis, and diagnostic pigment identification via high-performance liquid chromatography (HPLC) for analysis of photopigments diagnostic of algal community composition. Pigment extraction was performed by submerging filters of concentrated subsamples from bioassays in MeOH, sonicating, and centrifugation. HPLC procedures are described in ref 47. Major phytoplankton taxa and diagnostic pigments included chlorophytes or “green algae” (chlorophyll b, lutein, violaxanthin), diatoms (fucoxanthin) cyanobacteria (zeaxanthin, myxoxanthophyll, echinenone) and dinoflagellates (peridinin) using *Shimadzu’s LabSolutions Lite* software, and in total 19 distinct diagnostic pigments were identified and quantified via a calibration curve generated from commercially available standards (DHI, Denmark). Diagnostic photopigments were run through a matrix factorization program, ChemTax, to normalize pigment (carotenoids, Chl b)-specific biomass for each major phytoplankton group (cyanobacteria, chlorophytes, cryptophytes, diatoms, and dinoflagellates) as a fraction of total phytoplankton biomass, Chl a, as detailed in refs 48 and 49. Members of major groups were confirmed manually using microscopic imaging.

The raw data obtained from HPLC were statistically analyzed and subsequently converted into bar graphs to enhance the visual representation of the findings.

Soluble nutrient concentrations on precombusted GF/F filtered water were determined utilizing a LACHAT QuikChem 8000 Flow Injection Analysis System following LACHAT Instruments’ QuikChem Method 10-107-04-3-P for total dissolved nitrogen (TDN). The method detection limit for this analyzer is 93.34 μg/L for TDN. A SEAL QuAAtro39 Continuous Segmented Flow Analyzer (QuAAtro SEAL Analytical Inc.) was utilized following SEAL method guidelines in accordance with standard U.S. EPA methods to determine concentrations of phosphate (PO_4_) (Method no. Q-037-05 Rev. 4), silica (SiO_2_) (Method no. Q-005-04 Rev. 2), ammonium (NH_4_) (Method no. Q-033-04 Rev. 8) and nitrate/nitrite (NO_3_/NO_2_) (Method no. MT3B Q-035-04 Rev 10). Detection limits for this analyzer are as follows: 6.25 μ g/L NO_*x*_, 1.49 μ g/L PO_4_, 3.14 μ g/L NH_4_, and 51.31 μ g/L SiO_2_.

Particulate organic carbon (PC) and nitrogen (PN) were measured on seston collected on precombusted GF/F filters, analyzed by high-temperature combustion using a Costech ECS 4010 analyzer.^50^ DIC and DOC were measured on a Shimadzu total organic carbon analyzer (TOC-5000A).^51^

## 3 Results and Discussion

### 3.1 Biological Treatment Performance

Table S4 provides the detailed influent and effluent characteristics of SBRs R1 and R2 for biological nitrogen removal. Total nitrogen (TN) concentrations were notably high in the influents, with R1 at 55.6 mg/L and R2 at 90.7 mg/L, which were reduced to 6.8 mg/L (88% removal) in R1 and 8.2 mg/L (91% removal) in R2 effluents. Ammonium nitrogen (NH_4_^+^-N) concentrations showed a substantial decrease, with R1 reducing from 44.8 mg/L to 0.6 mg/L (99% removal) and R2 from 80.9 mg/L to 0.7 mg/L (99% removal), along with significant postnitrification nitrate-nitrogen (NO_3_–N) at 30.8 mg/L in R1 and 44.2 mg/L in R2, indicating effective nitrification and nitrogen assimilation. Effluents NO_3_–N concentrations were insignificant, with 0.73 mg/L in R1 and 1.06 mg/L in R2, indicating effective denitrification. Nitrite nitrogen (NO_2_–N) was negligible throughout the SBR treatments.

Dissolved organic nitrogen (DON), representing the organic nitrogen content, was less effectively removed compared to inorganic nitrogen. In R1, DON decreased from 9.8 mg/L in the influent to 5.5 mg/L in the effluent (44% removal), while in R2, it reduced from 9.3 mg/L to 6.5 mg/L (30% removal). This comparison highlighted the SBR’s higher efficiency in removing inorganic nitrogen species, particularly ammonium, but limited effectiveness in reducing organic nitrogen, which persisted in the biological treatment processes. Limited studies provide information on specific DON removal from leachate-wastewater biological cotreatment making it difficult to compare the results obtained in this study. One study showed that over 70% DON was removed during biological treatment at a local wastewater treatment plant, which does not receive any landfill leachate.^52^ Another study evaluated 11 different leachates discharged into local wastewater treatment plants at 10% and 1% volumetric contribution, and its effect on wastewater effluent DON; however it failed to provide the actual percentage removal of DON after biological treatment.^35^

Other parameters also indicated effective treatment in the SBR. Chemical oxygen demand (COD) was reduced from 1136 mg/L to 279 mg/L in R1 (76% removal) and from 646 mg/L to 258 mg/L in R2 (60% removal), showing substantial removal of organic content. Biological oxygen demand (BOD) similarly decreased, with R1 showing a reduction from 409 mg/L to 86 mg/L (79% removal) and R2 from 220 mg/L to 73 mg/L (67% removal). Alkalinity decreased significantly, from 202 mg/L to 35 mg/L in R1 and from 216 mg/L to 32 mg/L in R2, reflecting alkalinity consumption during the nitrification process. Phosphorus levels were also reduced, with R1 decreasing from 31.6 mg/L to 16.5 mg/L (48% removal) and R2 from 38.6 mg/L to 14.2 mg/L (63% removal), indicating that phosphorus was not a limiting factor throughout the biological treatment. In summary, the SBR treatment effectively reduced inorganic nitrogen (especially ammonium), COD, BOD, and phosphorus levels, indicating successful removal of these nutrients and contaminants. However, the lower removal efficiency of DON highlighted the challenge of reducing organic nitrogen, suggesting that additional treatment steps might have been necessary to address persistent DON in the effluents.

### 3.2 Physicochemical Treatment Performance

Figure 1 illustrates the changes in nitrogen species, including dissolved organic nitrogen (DON), nitrite (NO_2_^–^-N), nitrate (NO_3_^–^-N), and ammonium (NH_3_^+^-N), before and after the Fenton treatment under the optimum condition (Figures S3 and S4). For R1, the total nitrogen decreased from 5.9 mg/L before treatment to 3.69 mg/L after treatment. DON, the dominant nitrogen species, was significantly reduced from 4.72 mg/L to 2.18 mg/L. NH_3_–N increased from 0.44 mg/L to 1.03 mg/L, and NO_3_–N slightly decreased from 0.73 mg/L to 0.48 mg/L. NO_2_–N levels remained consistently low. In the case of R2, total nitrogen dropped from 7.36 mg/L to 4.32 mg/L following Fenton treatment. The DON decreased notably from 6.04 mg/L to 2.41 mg/L. NH_3_–N levels rose from 0.19 mg/L to 1.39 mg/L, while NO_3_–N decreased slightly from 1.12 mg/L to 0.52 mg/L. Overall, the Fenton treatment led to a significant reduction in DON for both effluents, which contributed to the decrease in total nitrogen. However, there was a significant increase of NH_3_–N and/or NH4^+^-N for both cases, indicating conversion of organic nitrogen to ammonium nitrogen. This suggests that while Fenton treatment effectively reduced DON, it may lead to the conversion of DON to ammonia/ammonium nitrogen, indicating Fenton treatment is a potential pretreatment of nitrification processes toward more effective total nitrogen removal. Another study on the use of Fenton treatment shows potential for partial degradation of DON in wastewater, achieving around 25% DON removal at an optimal hydrogen peroxide dose.^53^ Due to a lack of any studies on the Fenton treatment for DON removal from landfill leachate–wastewater cotreatment, it was difficult to compare the performance results with this study.

**Figure 1:**
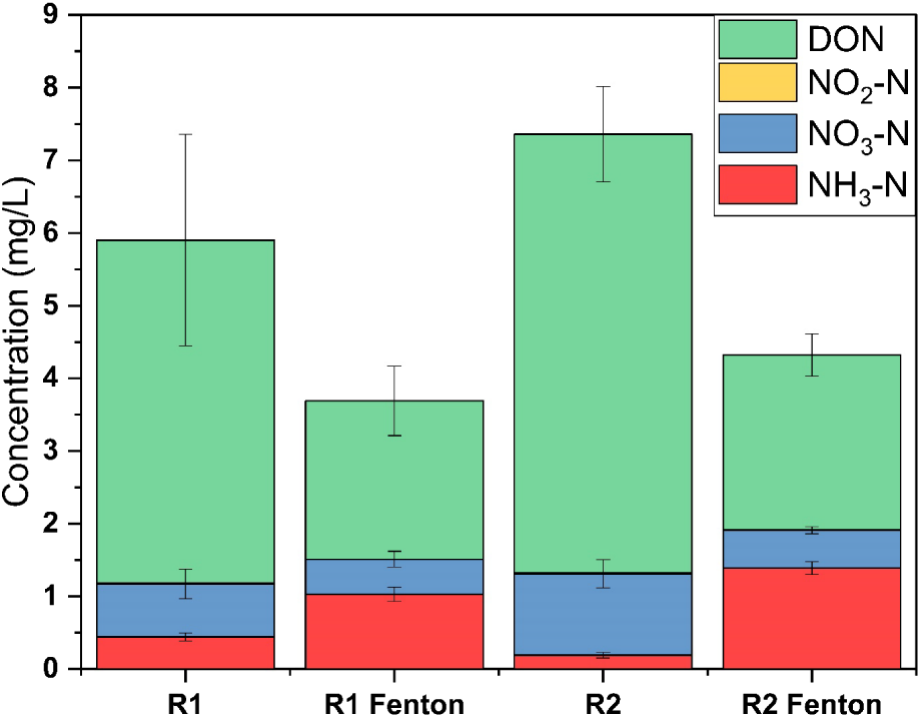
Nitrogenous species in R1 and R2 effluents before and after Fenton treatment.

Figure 2 shows the concentrations of various nitrogen species—dissolved organic nitrogen (DON), nitrite (NO_2_^–^-N), nitrate (NO_3_^–^-N), and ammonia (NH_3_–N)—before and after granular activated carbon (GAC) adsorption for R1 and R2 effluents at the optimum condition (Figures S4 and S5). Total nitrogen (TN) concentrations are indicated for both influent and treated samples. For R1 effluent, the total nitrogen concentration was 4.6 mg/L before GAC adsorption, with DON comprising the majority at 3.86 mg/L. Following the GAC treatment, total nitrogen significantly decreased to 0.8 mg/L. DON levels also dropped remarkably to 0.18 mg/L, while NH_3_–N and NO_3_–N levels decreased slightly to 0.62 mg/L and 0.54 mg/L, respectively. Notably, NO_2_–N levels remained negligible both before and after treatment. For R2 effluent, total nitrogen concentration before GAC adsorption was 6.97 mg/L, with DON accounting for 5.73 mg/L. After GAC adsorption, total nitrogen was reduced to 0.76 mg/L, with DON decreasing to 0.95 mg/L. NH_3_–N and NO_3_^–^-N concentrations after GAC adsorption were also relatively low, at 0.44 mg/L and 0.12 mg/L, respectively. Overall, the GAC adsorption process demonstrated a significant reduction in total nitrogen concentrations for both R1 and R2 effluents, particularly DON, which was the predominant nitrogen species. These results indicate that GAC effectively removes all nitrogen species, highlighting its potential as a treatment option for reducing nitrogen levels in wastewater. To compare the performance of DON removal in this study with previous findings, an extensive literature review was carried out, but no study provided specific GAC treatment performance for DON removal. Few studies used different adsorbent materials such as Al-pillared bentonite, layered double hydroxides (LDH), and nanomaterials for DON removal, but only studied the dynamics behind the DON removal without any treatment performance evaluation, especially for the biological treated leachate-wastewater mix.^54–58^ The adsorption isotherms for GAC are discussed in the Supporting Information with Figures S6–S9, Tables S6 and S7.

**Figure 2:**
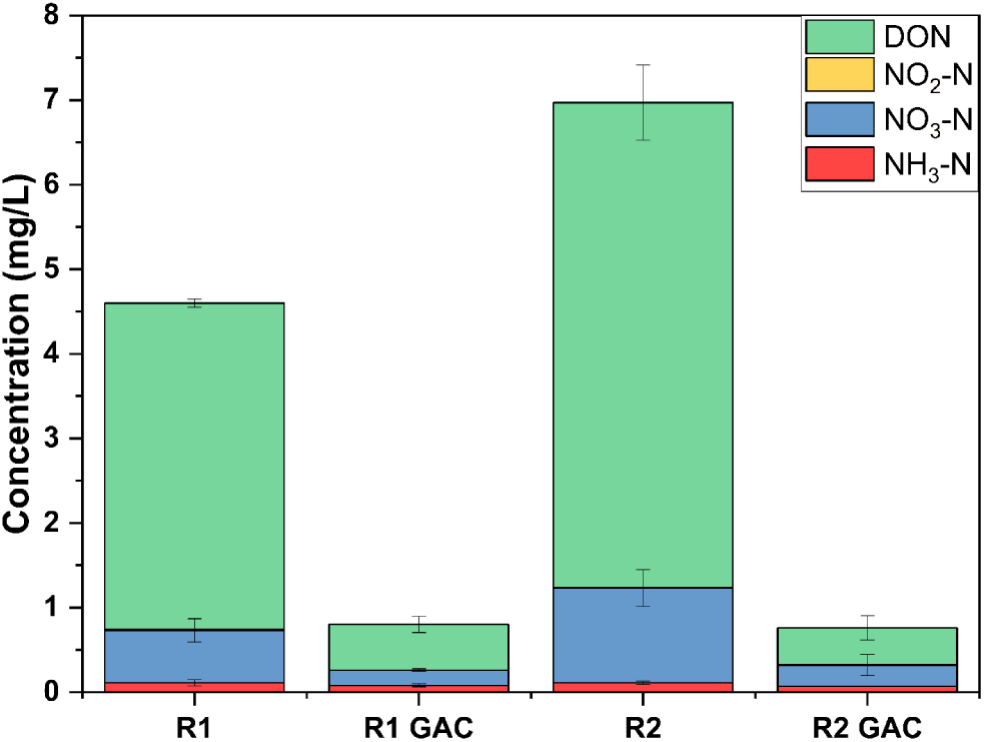
Nitrogenous species in R1 and R2 effluents before and after GAC treatment.

### 3.3 Dissolved Organic Matter (DOM) Spectral Characterization

#### 3.3.1 Fourier-Transform Infrared (FTIR) Spectroscopy

FTIR spectroscopy provided valuable insights into the functional groups present in the samples from various treatment stages, including high and low organic leachate, wastewater, and treated samples: R1, R1-Fenton, R1-GAC, R2, R2-Fenton, and R2-GAC. The FTIR spectra for R1 and R2 effluents before and after Fenton and GAC treatments are shown in Figure 3. The FTIR spectra for the high and low organic leachate and wastewater are shown in Figure S10. The FTIR peaks listed in Table S5 were characterized based on previously published studies on organic matter characterization in wastewater and landfill leachate (Bolyard et al., 2019; Coates, 2000). The analysis highlighted how different treatment methods influenced functional group composition. The presence of primary amides was observed through N–H stretching, with R1 exhibiting a peak at 3406 cm^–1^ and R2 showing a peak at 3425 cm^–1^. The slight variation in wavenumbers suggests differences in the amide concentration or environment, which is likely due to the higher organic content in R1. Additionally, C=N stretching for imines/oximes appeared at 1662 cm^–1^ in R1 and 2924 cm^–1^ in R2, indicating different nitrogenous compound profiles reflective of their source leachates.

**Figure 3:**
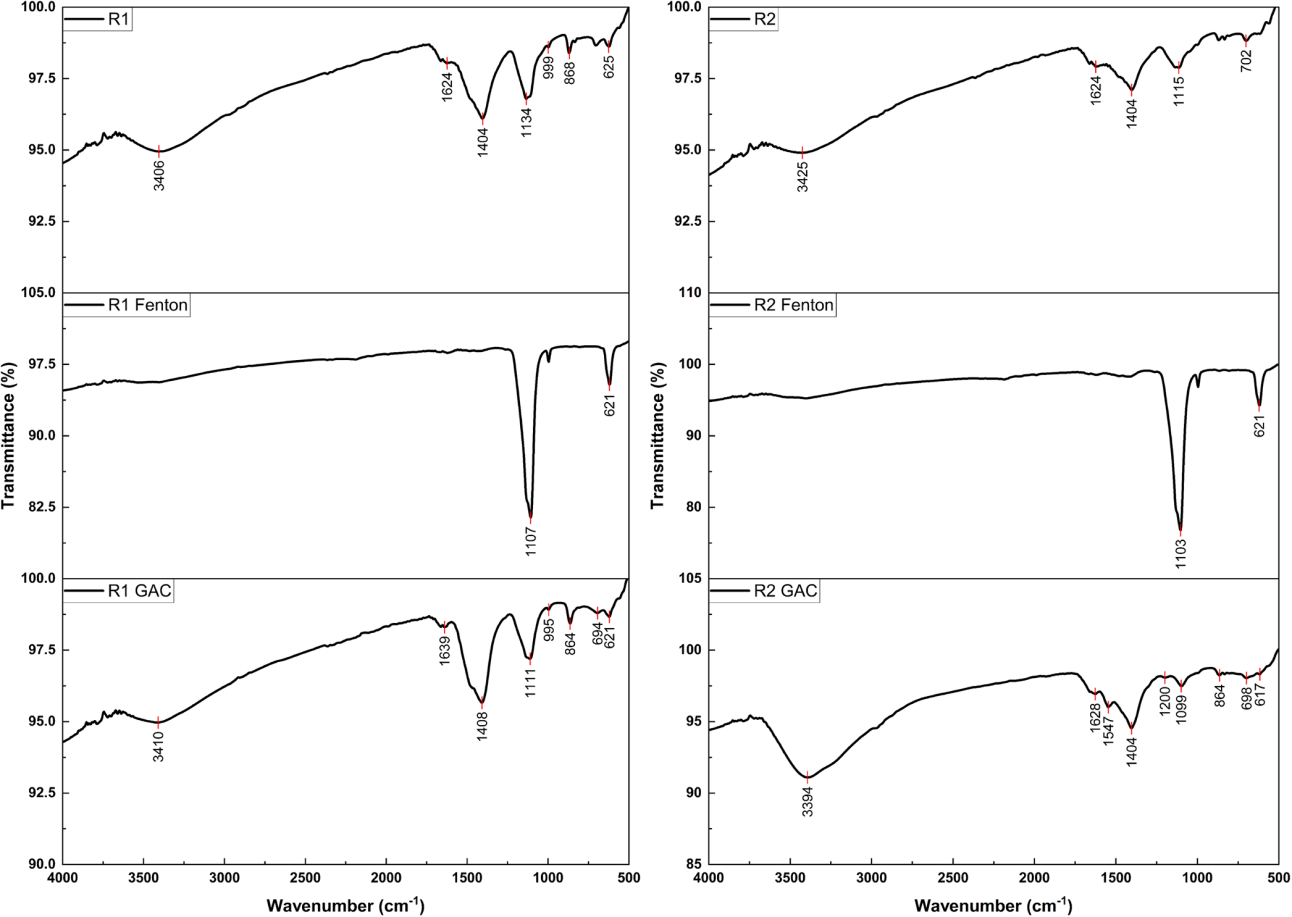
FTIR spectra for R1 and R2 effluents before and after Fenton and GAC treatment.

Following the Fenton treatment, the primary amides in R1-Fenton disappeared, demonstrating effective degradation due to the oxidative process. Both R1-Fenton and R2-Fenton also exhibited significant shifts in the S–O stretching bands (1107/621 cm^–1^ for R1-Fenton and 1103/621 cm^–1^ for R2-Fenton), suggesting transformations in the sulfate compounds, likely due to partial oxidation. Despite the effectiveness of the Fenton treatment, organic nitrates persisted in both samples, as indicated by peaks around 1624 cm^–1^, suggesting that certain nitrogenous substances resist complete breakdown. Post-GAC treatment, the N–H stretching of primary amides reappeared in R1-GAC (3410 cm^–1^) and R2-GAC (3394 cm^–1^), indicating that GAC primarily adsorbed these compounds without chemically altering them. Organic nitrates remained detectable, with peaks at 1639 cm^‑1^ in R1-GAC and 1628 cm^–1^ in R2-GAC, reflecting the persistence of some nitrogenous compounds even after GAC treatment. Differences in nitrate/nitrite peak positions (1408 cm^–1^ for R1-GAC and 1404 cm^–1^ for R2-GAC) further revealed slight variations in residual nitrogenous compounds.

The S–O stretching bands in R1-GAC (1111/621 cm^–1^) and R2-GAC (1099/617 cm^–1^) exhibited shifts, indicating that while GAC treatment affected sulfate-related structures, these compounds were not completely removed. Overall, FTIR analysis revealed a complex interaction between the functional groups during the treatment processes. The presence and behavior of primary amides, nitrogenous compounds, and sulfate groups varied significantly between samples and treatments, emphasizing that high organic leachate (R1) required more intensive treatment for the effective removal of these functional groups compared to low organic leachate (R2). Understanding these dynamics is essential for optimizing wastewater treatment strategies.

#### 3.3.2 Nuclear Magnetic Resonance (NMR) Spectroscopy

The nuclear magnetic resonance (NMR) analysis provided fundamental structural insights into the organic materials present in the samples. The ^13^C NMR spectra were partitioned into eight distinct regions of chemical shifts associated with specific functional groups in accordance with previous studies on dissolved organic matter (DOM) in natural water, wastewater, and landfill leachates.^59–64^ These functional groups and their corresponding shifts were used to characterize the chemical composition before and after treatment.

Multiple peaks were observed in the ^13^C NMR spectra (Figures 4 and S11) for the high-organic leachate, low-organic leachate, and wastewater, indicating the presence of diverse DOM. In the alkyl carbon region, two peaks at 10 and 13 ppm were detected across all the samples, with a moderate peak at 19 ppm present in both leachates, suggesting alkyl carbon structures. Notable peaks appeared at 30–31 and 23 ppm, corresponding to methylene (−CH_2_) and methyl (−CH_3_) groups, respectively, which are commonly found in proteinaceous materials and long fatty acid chains.^65,66^

**Figure 4:**
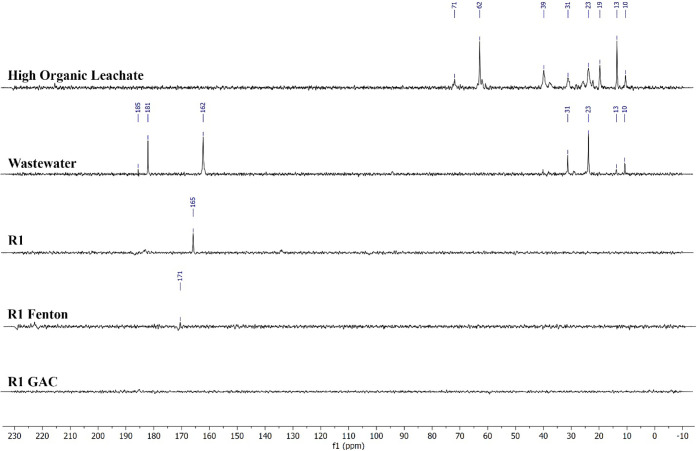
^13^C NMR spectra for high organic leachate, wastewater, and R1 effluent before and after the Fenton and GAC treatment.

A distinct peak at 37 ppm was detected in the low organic leachate, while a peak at 39 ppm appeared in the high organic leachate, which could be attributed to quaternary carbons or methylene carbons linked to nitrogen (N–CH_2_).^60,61^ Previous studies on landfill leachate DOM have reported a similar peak at 37 ppm, although some investigations have not observed this.^67,68^ Additionally, minor peaks at 44 and 61 ppm were found in the low organic leachate, whereas a more prominent peak at 62 ppm was observed in the high organic leachate, likely derived from methoxyl groups associated with lignin, hemicellulose, or peptide structures.^69–71^ High organic leachate showed a significant O-alkyl C peak at 71 ppm, indicating the presence of carbohydrates or carbohydrate-like substances within DOM. This peak has often been associated with cellulose residues in various studies.^69,72^ The aromatic region (129–138 ppm) displayed moderate peaks in low-organic leachate, which may correspond to oxygen- or nitrogen-substituted aromatic carbons, phenolic structures, or lignin derivatives.^65,66,70^ Additionally, a COO/N–C=O signal characteristic of protein-like materials was detected at 175 ppm in low-organic leachate, suggesting that carboxyl or ester groups are possibly derived from uronic acids or suberin.^71^ The carbonyl signals in the 160–185 ppm range showed considerable variation across the samples. Peaks at 162 ppm were detected in the wastewater, whereas peaks at 181–185 ppm were prominent in low organic leachate and wastewater, indicating the presence of carbonyl C in carboxylic, ester, or amide compounds.^65,72^ The region between 220 and 191 ppm exhibited no signals, suggesting a negligible or absent carbonyl C from ketones or aldehydes.

Biological treatment effectively removed most organic peaks as shown in the spectra of R1 and R2, leaving only a residual peak at 164–166 ppm. This indicated that most of the organic components, particularly nitrogenous and carbonyl-containing compounds, were significantly reduced during the process. Fenton oxidation further degraded organic matter in R1 and R2. In the R1-Fenton process, the elimination of the peak at 165 ppm suggests the efficient oxidation of carbonyl-containing compounds. A new minor peak at 171 ppm was observed, possibly owing to the carboxylic groups of uronic acids or other oxidation byproducts.^69^ In contrast, the R2-Fenton spectrum displayed no detectable peaks, indicating that Fenton treatment was highly effective in removing organic substances from the low-organic-leachate-derived sample. GAC treatment demonstrated significant efficacy in removing residual DOM from both the R1 and R2 samples. All peaks were eliminated in the spectra of R1-GAC and R2-GAC, indicating complete removal of organic matter. However, in R2-GAC, a low-intensity peak persisted at 165 ppm, suggesting incomplete removal of certain carbonyl-containing compounds.

NMR analysis provides valuable insights into the complex nature of DOM present in landfill leachates and wastewater. The high organic leachate exhibited a greater variety of peaks, reflecting higher concentrations of nitrogenous and carbonyl compounds. In contrast, low-organic leachate displayed fewer peaks, indicating a lower concentration of organic matter. The results showed that biological treatment, Fenton oxidation, and GAC treatment played crucial roles in transforming or removing various organic compounds, with Fenton treatment displaying high efficacy in degrading carbonyl compounds, whereas GAC treatment effectively adsorbed residual organic nitrogen species.

### 3.4 Algal Bioassay

The algal bioassay analysis of R1 and R2 effluents treated separately with Fenton and GAC methods provides a comprehensive overview of the concentration trends of various diagnostic algal pigments over time, including total chlorophyll a, chlorophyll b, fucoxanthin, myxoxanthophyll, peridinin, zeaxanthin, and alloxanthin. Figure 5 shows the temporal trend for Total chlorophyll A (all algae) and Figure S11 shows the temporal trend for total chlorophyll B (chlorophytes), fucoxanthin (diatoms), myxoxanthophyll (cyanobacteria), peridinin (dinoflagellates), zeaxanthin (cyanobacteria), and alloxanthin (cryptophytes).

**Figure 5:**
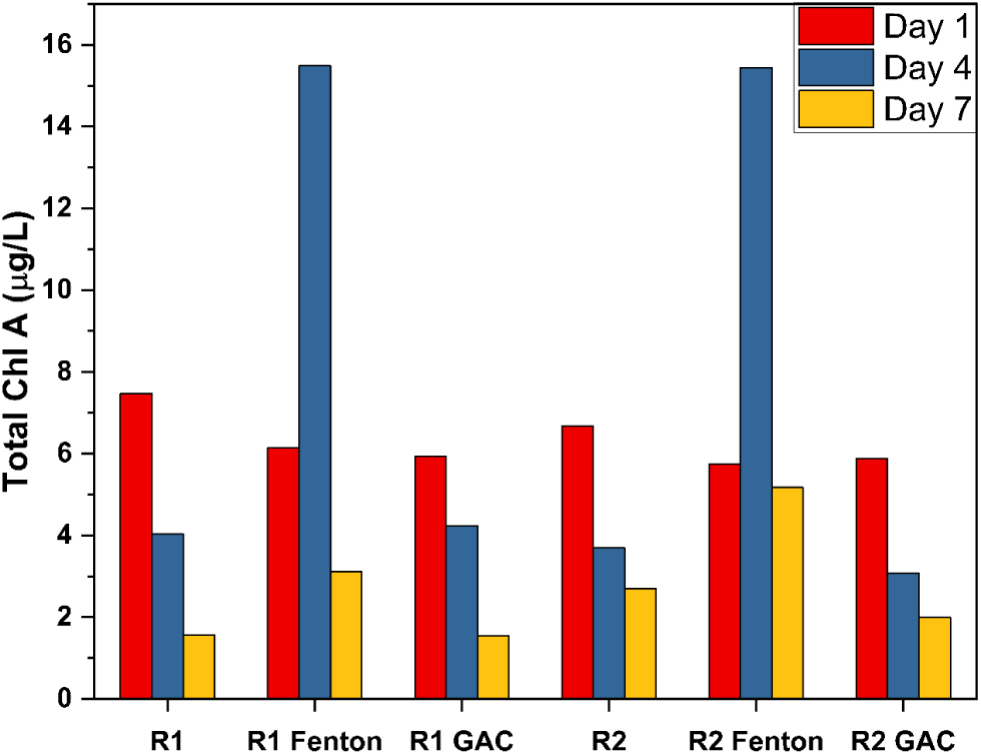
Temporal trend in total chlorophyll A concentration in R1 and R2 effluents before and after the Fenton and GAC treatment.

The total chlorophyll A concentrations revealed significant differences based on the treatment methods and time. For R1, the Fenton treatment increased total chlorophyll A concentration from 6.15 μg/L on day 1 to 18.28 μg/L by day 4, indicating initial enhancement of algal growth due to rDON conversion to ammonium by Fenton oxidation as shown in Figure 1. However, this was followed by a decline to 4.69 μg/L by day 7, suggesting nutrient depletion. Conversely, GAC treatment for R1 showed a decrease from 5.94 μg/L on Day 1 to 4.55 μg/L by Day 4 and further reduced to 4.24 μg/L by Day 7, indicating that GAC adsorption may have removed all N species and limited the availability of nutrients for algal growth. For R2, the Fenton treatment also showed an upward trend, increasing from 5.75 μg/L on Day 1 to 16.76 μg/L by Day 4, followed by a decrease to 9.50 μg/L on Day 7. Similar to R1, this pattern indicated a temporary stimulation of algal growth, likely influenced by initial nutrient availability due to rDON conversion to ammonium, before stabilizing at a lower concentration. In contrast, GAC treatment for R2 resulted in a more pronounced decline, from 5.88 μg/L on Day 1 to 3.90 μg/L on Day 4 and stabilizing at 3.08 μg/L by Day 7, suggesting that GAC may adsorb various nitrogen species and inhibit algal growth. The results of the other six algal bioassay pigments have been discussed in the Supporting Information with Figure S12.

Overall, the bioassay data indicate that both treatment methods have distinct effects on algal pigment concentrations, with Fenton treatment generally promoting initial algal growth, particularly for total chlorophyll A, fucoxanthin (diatoms), and myxoxanthophyll (cyanobacteria), while GAC treatment appears to inhibit total algal growth. The variation in pigment concentrations over time reflects the complex interactions between the treatment methods and algal community dynamics, providing insights into optimizing treatment strategies for enhanced algal biomass control in effluent management.

This study builds on previous work that assessed the bioavailability of DON fractions using algal bioassays with resin-extracted samples.^73^ Also, it shows novel results that complement a previous study that assessed the bioavailability of fractionated effluent DON using *Pseudokirchneriella subcapitata* in nutrient-depletion bioassays.^74^ While these studies quantified bioavailable N and P using growth-based standard curves under controlled nutrient conditions, the current approach in this study focused on practically used effluent treatments (Fenton and GAC) and their impact on algal pigment dynamics in natural algal communities. Unlike fractionation-based methods, our bioassays directly captured community-level algal responses to residual DON in treated leachate effluents, offering practical insight into the ecological impacts of advanced treatment strategies.

### 3.5 Environmental Implications

This study demonstrates that Fenton treatment effectively removes bulk organic matter and inorganic nitrogen species while converting refractory dissolved organic nitrogen (rDON) to ammonium, enhancing its biodegradability and bioavailability. Fenton oxidation applied as a pretreatment can enhance subsequent biological processes by converting recalcitrant organic nitrogen into biodegradable ammonium, thereby mitigating nutrient discharge into surface waters, whereas postbiological Fenton treatment may increase nutrient bioavailability and potentially stimulate algal growth in receiving ecosystems. A previous study emphasized the potential of Fenton treatment for degrading refractory organic nitrogen in landfill leachate but noted the necessity of follow-up biological treatment for complete nitrogen removal.^75^ Therefore, Fenton oxidation is recommended as a cost-effective pretreatment for landfill leachate management.

Granular activated carbon (GAC) is highly effective for treating landfill leachate, particularly in removing refractory organic matter and organic nitrogen that remain after biological processes. Its extensive surface area and porous structure facilitate the adsorption of organic pollutants, including hydrophobic and low-molecular-weight compounds, contributing to the mitigation of eutrophication risks. However, GAC treatment has notable limitations. Competitive adsorption can reduce its efficiency, prioritizing the removal of simpler compounds over refractory ones. Additionally, GAC’s adsorption capacity diminishes over time, necessitating frequent and costly regeneration or replacement, which is energy-intensive and may produce secondary waste requiring further management. GAC is also less effective for highly polar or water-soluble contaminants, leaving some pollutants untreated.

Cost analyses, such as the study by Oloibiri et al., reveal that GAC significantly increases overall treatment expenses, particularly when combined with nitrification-denitrification, as GAC contributes most to the cost.^76^ Modifying biological treatments can reduce GAC-related expenses, but secondary waste generation remains a challenge. Despite its effectiveness for refractory contaminants, GAC’s cost and sustainability concerns warrant careful integration into landfill leachate treatment frameworks.

## 4 Conclusion

•Both Fenton oxidation and granular activated carbon (GAC) adsorption significantly reduced refractory dissolved organic nitrogen (rDON) in biologically treated landfill leachate and sewage mixtures, with GAC achieving higher removal efficiencies (86%–92%) compared to Fenton treatment (52%–60%).

•Fenton and GAC bear different rDON removal mechanisms. Fenton treatment converts rDON into ammonium nitrogen (NH_4_^+^-N), enhancing biodegradability and potentially serving as an effective pretreatment step for nitrification-based total nitrogen removal. GAC adsorption operates through nonselective physical adsorption, removing all nitrogen species without altering their chemical structure.

•Fenton and GAC have different impacts on algal growth. Fenton treatment, by increasing NH_4_^+^-N levels, temporarily stimulated algal growth as reflected by higher chlorophyll A concentrations in bioassays, underscoring the need for subsequent nutrient management. GAC treatment effectively reduced bioavailable nitrogen supporting algal growth, resulting in stable and/or declining chlorophyll A levels over time.

•Fenton and GAC have different practical considerations. Manufacturing GAC involves high energy and/or chemical consumption, and carbon emission, which has positive environmental impacts. GAC treatment requires frequent regeneration or replacement, which increases operational costs and generates secondary waste, necessitating sustainable management strategies. Fenton treatment’s chemical demand and potential secondary nutrient enrichment in effluents make it more suited as a pretreatment rather than a standalone solution.

## Supplementary Material



## Notes

Additional details of bioreactor operation, wastewater characteristics and spectra, optimum condition of treatment technologies, adsorption isotherms, diagrammatic sketch of experimental setup, GAC characteristics, and *in situ* algal bioassay (PDF)
