# Supplementary material for: Advanced Treatment of Landfill Leachate Induced Dissolved Organic Nitrogen (DON) and Its Influence on the Estuarine Algal Community
Source: ACS ES T Water. 2025 Aug 19;5(9):5025–36. doi: 10.1021/acsestwater.4c01265 (PMC12439301; doi:10.1021/acsestwater.4c01265)
Supplement: Supplementary file 1 [file ew4c01265_si_001.pdf]

Advanced Treatment of Landfill Leachate Induced Dissolved Organic Nitrogen (DON) and its Influence on the Estuarine Algal Community

Harsh V. Patel[1], Md Redowan Rashid[1], Md Ashik Ahmed[2], Lifeng Zhang[2], Brian Brazil[3], Wenzheng Yu[4], Hans W. Paerl[5,*], Renzun Zhao[1,*]

[1]Department of Civil, Architectural, and Environmental Engineering, North Carolina A&T State University, Greensboro, NC, United States, 27411

[2]Joint School of Nanoscience and Nanoengineering, North Carolina A&T State University, Greensboro, NC, United States, 27401

[3]Waste Management, Inc., Gaithersburg, Maryland, United States, 20878

[4] State Key Laboratory of Environmental Aquatic Chemistry, Research Center for Eco-Environmental Sciences, Chinese Academy of Sciences, Beijing, China, 100084

[5]Institute of Marine Sciences, University of North Carolina at Chapel Hill, Morehead City, NC, United States, 28557

* Corresponding authors: rzhao@ncat.edu; hans_paerl@unc.edu

Table S1. Characteristics of raw sewage collected from the local wastewater treatment plant on different days of reactor operation. The unit of measurement is mg/L, and mg/L as $CaCO_3$ for alkalinity.

**Sewage Characteristics**	**Day**									
	**1**	**13**	**23**	**49**	**66**	**74**	**92**	**116**	**133**	**155**
**pH**	7.04	37.50	33.77	0.17	0.05	3.51	364.00	95.00	25.65	165.00
**TN**	7.10	35.60	31.89	0.74	0.04	2.93	341.00	86.00	27.44	171.00
**NH_4^+-N**	7.08	36.70	33.20	0.05	0.05	3.41	260.00	65.00	28.21	161.00
**NO_3^--N**	7.22	35.60	31.30	0.31	0.06	3.94	379.00	90.00	27.33	176.00
**NO_2^--N**	7.30	35.80	31.89	0.25	0.04	3.62	378.00	92.00	23.67	177.00
**DON**	7.12	34.40	31.09	0.35	0.04	2.92	368.00	89.00	24.50	176.00
**COD**	7.01	34.30	29.12	0.43	0.13	4.62	303.00	74.00	25.88	170.00
**BOD**	7.12	34.80	31.30	0.38	0.04	3.08	326.00	75.00	26.90	182.00
**Phosphorus**	7.11	35.60	29.45	0.44	0.07	5.64	309.00	76.00	27.12	165.00
**Alkalinity**	7.21	35.30	29.10	0.51	0.09	5.60	328.00	80.00	26.40	169.00

Table S2: Characteristics of raw leachate fed to reactors 1 (R1) and 2 (R2). The unit of measurement is mg/L, and mg/L as $CaCO_3$ for alkalinity.

**Characteristics**	**High Organic Leachate (R1)**	**Low Organic Leachate (R2)**
**pH**	5.8 ± 0.04	7.9 ± 0.07
**TN**	2433 ± 12	1898 ± 7
**NH_4^+-N**	1502.5 ± 17.5	1628.5 ± 18.5
**NO_3^--N**	78.1 ± 1.8	37.6 ± 0.6
**NO_2^--N**	1.9 ± 0.24	0.86 ± 0.09
**DON**	850.5 ± 8.5	231 ± 6.8
**COD**	107685 ± 68	16980 ± 60
**BOD**	26075 ± 36	4152 ± 13
**Phosphorus**	533.5 ± 6.3	469.5 ± 1.5
**Alkalinity**	7633 ± 14.6	3988 ± 11.5

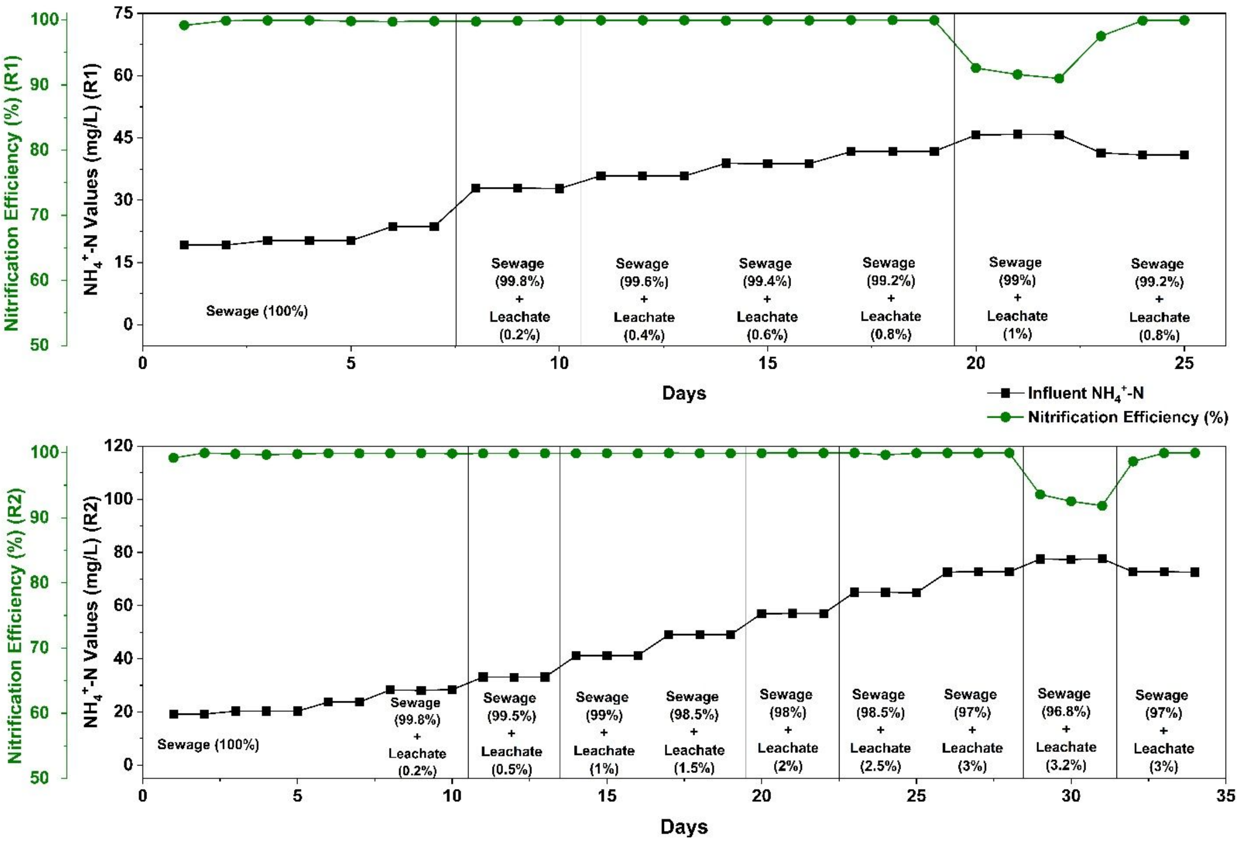

Figure S1: Nitrification for different landfill leachate and sewage ratios in the reactors.

Table S3: Performance of different operation cycles on nitrification, denitrification, and BOD removal

Operation Cycle	Nitrification	Denitrification	BOD Removal
**8 Hrs** (4hrs, 3hrs, 1hr)	76%	91%	43%
**12 hrs** (7.5 hrs, 3 hrs, 0.5 hr, 1 hr)	99%	85%	57%
**12 hrs** (7 hrs, 3.5 hrs, 0.5 hr, 1 hr)	99%	97%	57%
**12 hrs** (6.5 hrs, 3.5 hrs, 1 hr, 1 hr)	99%	97%	72%

Table S4. Influent and effluent characteristics from SBRs for biological nitrogen removal.

Parameters	R1 Influent	R1 Effluent	R2 Influent	R2 Effluent
**pH**	7.1 ± 0.2	7.7 ± 0.4	7.2 ± 0.1	7.6 ± 0.4
**TN (mg/L)**	55.6 ± 5.5	6.8 ± 1.9	90.7 ± 4.6	8.2 ± 1.5
**NH_4^+-N (mg/L)**	44.8 ± 2.4	0.6 ± 0.5	80.9 ± 2.2	0.7 ± 0.3
**NO_3-N (mg/L)**	0.9 ± 0.33	30.8±3.4 (post-nitrification) 0.7 ± 0.20 (post-denitrification)	0.4 ± 0.3	44.2±2.15 (post-nitrification) 1.1 ± 0.15 (post-denitrification)
**NO_2-N (mg/L)**	0.09 ± 0.01	0.003 ± 0.002	0.07 ± 0.005	0.008 ± 0.003
**DON (mg/L)**	9.8 ± 1	5.5 ± 1.2	9.3 ± 1.1	6.5 ± 1.1
**COD (mg/L)**	1136 ± 42	279 ± 110	646 ± 24	258 ± 80
**BOD (mg/L)**	409 ± 50	86 ± 35	220 ± 28	73 ± 40
**Alkalinity (mg/L as $CaCO_3$)**	202 ± 16	35 ± 4	216 ± 21	32 ± 8
**Phosphorous (mg/L)**	31.6 ± 3.3	16.5 ± 3	38.6 ± 6.5	14.2 ± 2

Table S5. IR peaks and their related functional categories in landfill leachate [1]

Wavenumber (cm-^{1})	Functional Groups
**3700–3400**	O-H stretching of bonded and non-bonded hydroxyl groups
**3372–3381**	O-H and N-H stretching of acids, amides and amines
**3180–3090**	NH_2 stretch of primary amides
**2981**	C-H stretching of aromatic structures
**2931–2936**	C-H stretching of methylene and aliphatic structures (e.g. fatty acids, waxes)
**2850**	C-H stretching of methylene and aliphatic structures
**2590–2560**	S-H stretching of thiol groups
**1740–1700**	C=O of aldehydes, ketones, carboxylic acids, and esters
**1685–1630**	C=O, COO- stretching, C=C stretching of amide I, carboxylates, aromatic ring modes, and alkenes
**1654–1645**	C=O and C=C of amide I, carboxylates, aromatic ring modes, and alkenes
**1635**	O-H bending of absorbed water
**1600–1590**	C=C of aromatic skeletons
**1560, 1546**	N-H in plane of amides II
**1515–1505**	Aromatic skeleton of lignin from lignocellulosic materials
**1450–1410**	N/A of carbonate
**1430–1420**	COO-stretching of carbonate
**1384**	N-O stretching of nitrate
**1350–1250**	C-N of primary and secondary aromatic amines
**1320**	C-N stretching of aromatic primary and secondary amines

**1295**	C-N stretching of amides
**1265–1240**	C-O, C-N of carboxylic acids and amide III
**1250–900**	C-O-C, C-O, and C-O-P of polysaccharides
**1140–1080**	S-O stretching of sulfate
**1114**	C-O stretching of secondary alcohols and ethers
**875**	C-O in the plane of carbonate
**713**	C-O in the plane of carbonate
**706**	N-H in plane of amide
**680–610**	S-O bend of inorganic sulfates

1. The optimum condition for Fenton treatment

The selected dosage range of hydrogen peroxide (H_2O_2) was 50-400 mg/L, with molar ratios of H_2O_2 to Fe(II) at 1:1, 1:3, 1:5, 1:7, 1:9, 3:1, and 5:1, based on COD removal efficiencies from previous studies [2–5]. COD removal efficiency increased with higher H_2O_2 dosages, aligning with findings from [6], who reported improved wastewater treatment using the Fenton process. This can be attributed to enhanced hydroxyl radical (•OH) production, promoting organic compound degradation. In this study, 200 mg/L was optimum for R1 and R2. The influence of the H_2O_2/Fe(II) molar ratio on COD removal showed that ratios exceeding 1:1 led to decreased efficiency. At 3:1 and 5:1 ratios, COD removal remained unchanged, regardless of H_2O_2 dosage. Conversely, increasing the ratio from 1:1 to 1:9 enhanced removal efficiency, in agreement with studies by Ben et al. (2009) and Cheng et al. (2018). In this study, ratios of 1:5, 1:7, and 1:9 performed best, with 1:9 yielding the highest efficiency. However, higher ratios resulted in substantial iron sludge production, raising concerns about sludge management costs. Therefore, the optimum ratios were determined to be 1:5 for R1 and R2 balancing removal efficiency with sludge minimization. Contour plots were utilized to visualize optimum conditions for Fenton treatment, with dosage and molar ratio on the X and Y axes, and COD removal efficiency on the Z axis. For R1, Figure S2 indicated that a dosage of 200 mg/L and a 1:5 molar ratio achieved approximately 65% COD removal. Similarly, R2 reached about 78% removal under the same conditions as shown in Figure S2.

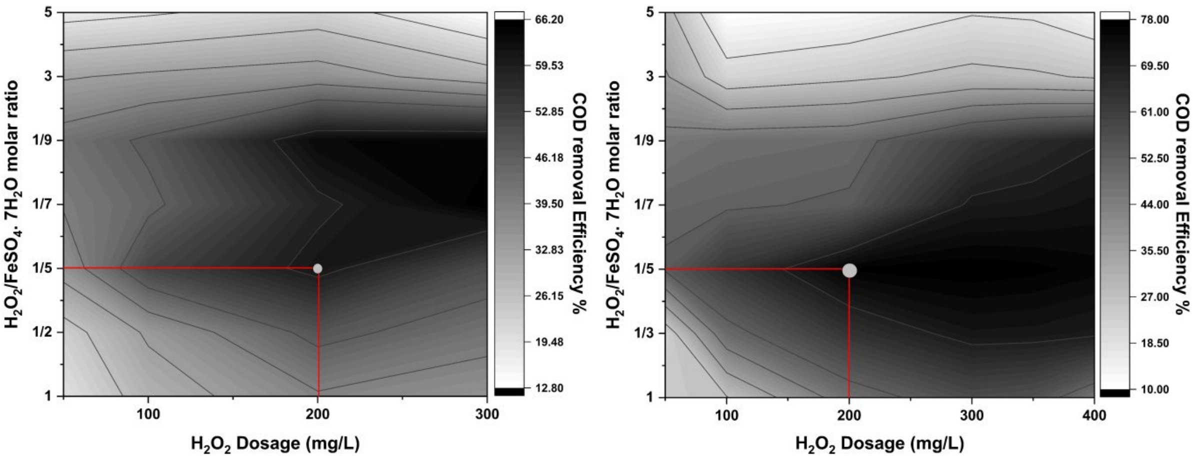

Figure S2: Optimum H_2O_2 dose and H_2O_2/Fe molar ratio for Fenton treatment for R1 (left) and R2 (right) based on COD removal.

Figure S3 shows the data plot for determining the optimum time for the Fenton reaction for R1 and R2, respectively. Duplicate experiments confirmed the reliability of these results, showing minimal variation. Most COD removal occurred within the first 10 minutes of the reaction across all influents, consistent with previous studies where 90-94% of COD was removed in the initial 10 minutes [3,7]. Therefore, a 10-minute reaction time was deemed optimum to maximize efficiency while conserving resources.

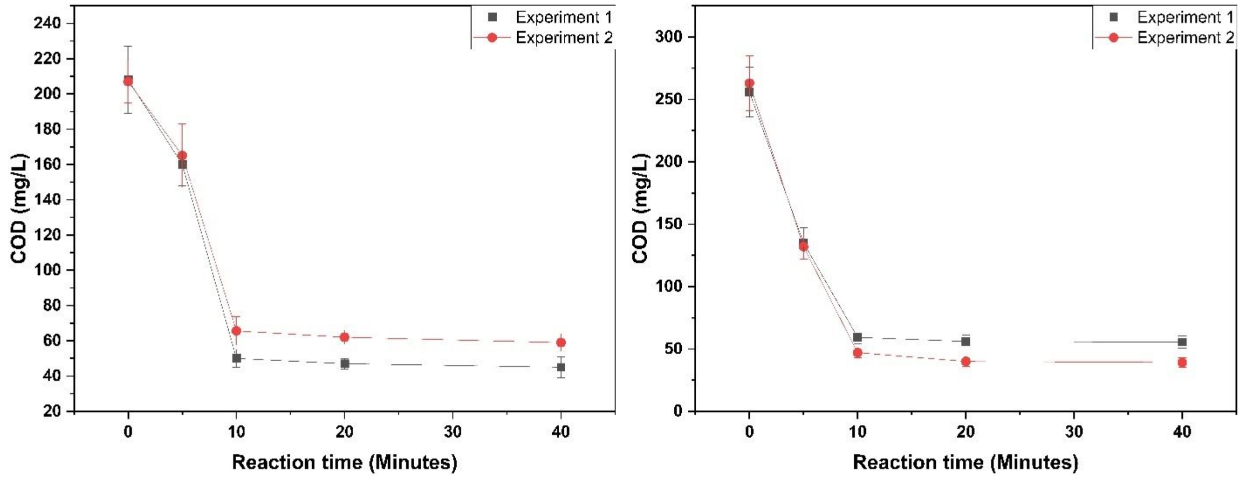

Figure S3: Optimum reaction time for Fenton treatment for R1 (left) and R2 (right). The error bar represents duplicate analysis.

In summary, optimum H_2O_2 dosages were 200 mg/L for R1 and R2, while the most effective molar ratios were 1:5 for R1 and R2. A 10-minute reaction time was sufficient for maximal COD removal. These results provide a comprehensive understanding of Fenton treatment, optimizing removal efficiency while minimizing reagent use.

2. The optimum GAC dose and contact time

The removal efficiencies of COD and DON showed a positive correlation with the dosage of GAC in both influents. The dosage was identified as optimal beyond which the elimination efficiencies of COD and DON exhibited minimal variation, indicating diminishing returns with further dosage increases. To validate our findings, duplicate experiments were conducted for each influent. In the case of R1, the optimal dosage for COD removal was determined to be 8–10 grams per liter (g/L), as shown in Figure S4. For DON, the optimal dosage was found to be 10 g/L, as shown in Figure S4. The data from both figures illustrate that the duplicate trials followed identical patterns for COD and DON removal. This consistency reinforces the reliability and robustness of the experimental results. Similarly, for R2, the optimal GAC dosage for COD removal was identified as 10 g/L (Figure S4), while the most effective dosage for DON was determined to be 7 g/L (Figure S4). As observed in R1, the duplicate experiments for R2 also produced identical patterns for COD and DON removal, further validating the consistency of the findings. These results underscore the effectiveness of GAC dosage optimization in removing

COD and DON from wastewater, while the duplicate trials highlight the reproducibility and reliability of the data. Duplicate tests were conducted to establish the optimal reaction time for granular activated carbon (GAC) treatment in each influent, similar to the methodology employed in the Fenton treatment. The minor variations observed in the results of the duplicate trials, as presented in Figure S4, contributed to the credibility and robustness of the research findings. Notably, the chemical oxygen demand (COD) values exhibited a rapid decline during the initial hours of the treatment process.

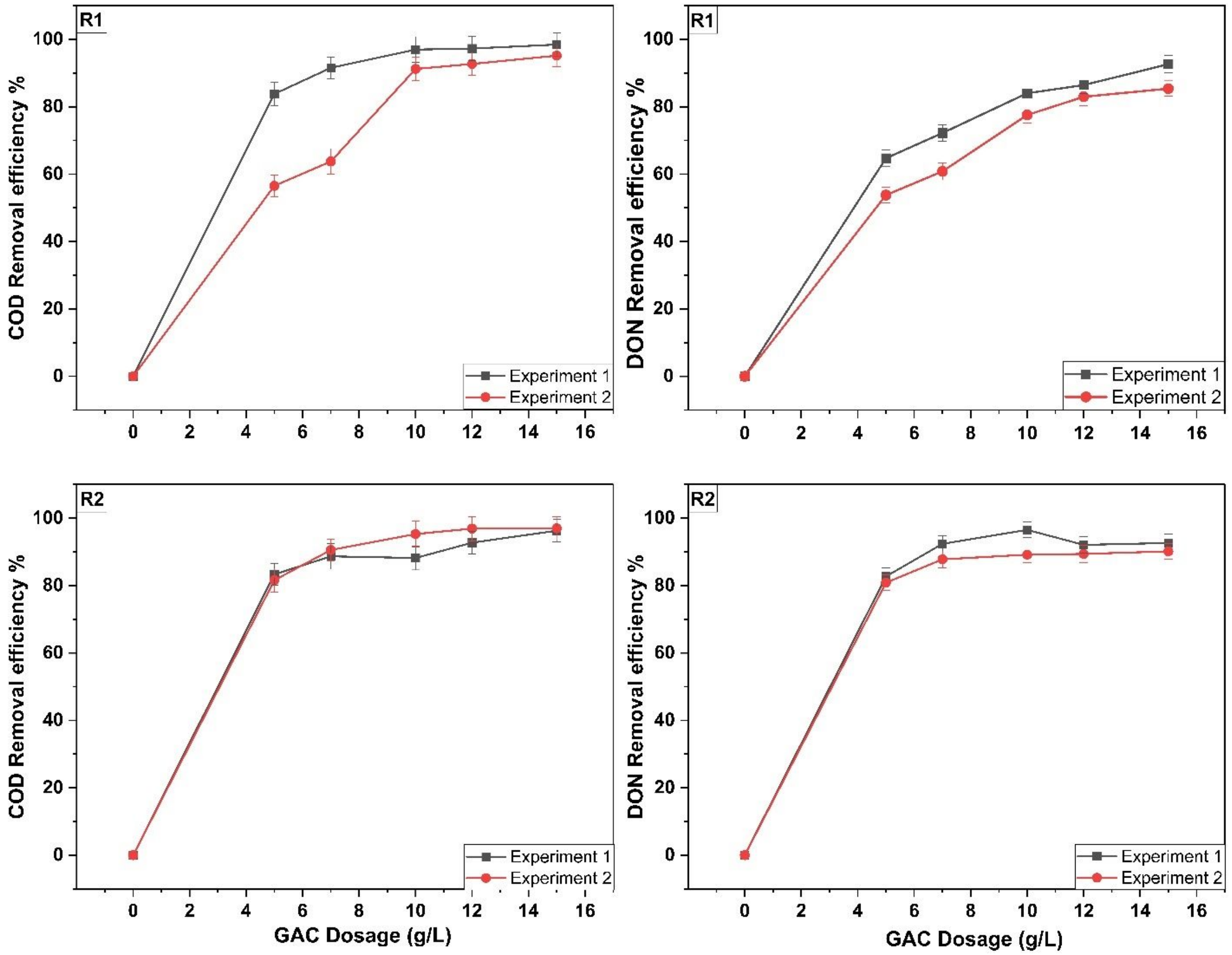

Figure S4: COD and DON removal from R1 and R2 from GAC treatment. The error bar represents duplicate analysis.

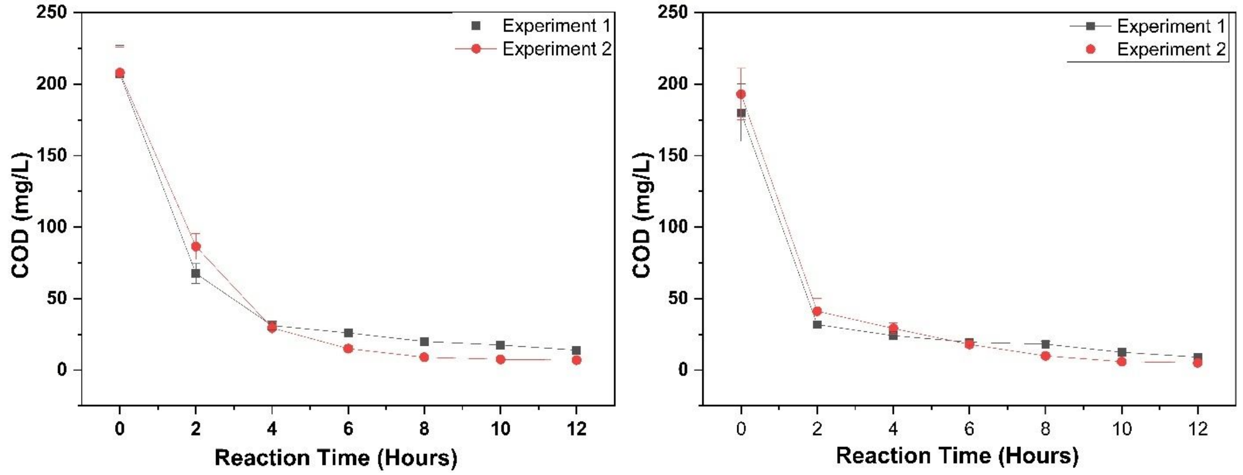

Figure S5: Optimum reaction time for GAC treatment for R1 (left) and R2 (right). The error bar represents the duplicate analysis.

The total reaction duration was 12 hours, during which the COD values remained relatively stable between the 6- and 12-hour marks. Therefore, a reaction time of 6 hours was selected for both influents to optimize treatment efficiency and conserve resources as shown in Figure S5.

3. Isotherm fitting for GAC Treatment

The maximum adsorption capacity was assessed by applying the Langmuir isotherm model, as illustrated in Figures S6 and S7. The slope and intercept values from the fitting curve are provided in Table S3. The Langmuir isotherm demonstrated a strong correlation with the experimental data, exhibiting r^2 values ranging from 0.92 to 0.99. These results indicated that the adsorption capacity for chemical oxygen demand (COD) was higher (42-45 mg/g) compared to that of dissolved organic nitrogen (DON) (0.5-1.3 mg/g). This phenomenon can be attributed to the greater initial concentration of COD relative to DON.

Table S3 further revealed that the adsorption capacity for COD was identical for both R1 and R2. In contrast, the adsorption capacity for DON in R2 (1.3 mg/g) was approximately three times greater than that observed in R1 (0.58 mg/g). Interestingly, R1 exhibited a higher maximum adsorption capacity (q_{max}) for COD compared to R2, while R2 demonstrated a greater q_{max} for DON than R1. This discrepancy may be linked to the initial concentrations of the respective species. Specifically, R1 had a higher initial COD than R2, which corresponded with a greater adsorption capacity. Conversely, R2 had a higher initial DON concentration than R1, resulting in an increased adsorption capacity for DON. Furthermore, the distinct compositions of organic compounds in the leachates from R1 and R2 could lead to varying affinities of the organic molecules for the surface of Granular Activated Carbon (GAC), contributing to the differences in GAC's adsorption capacities for COD and DON.

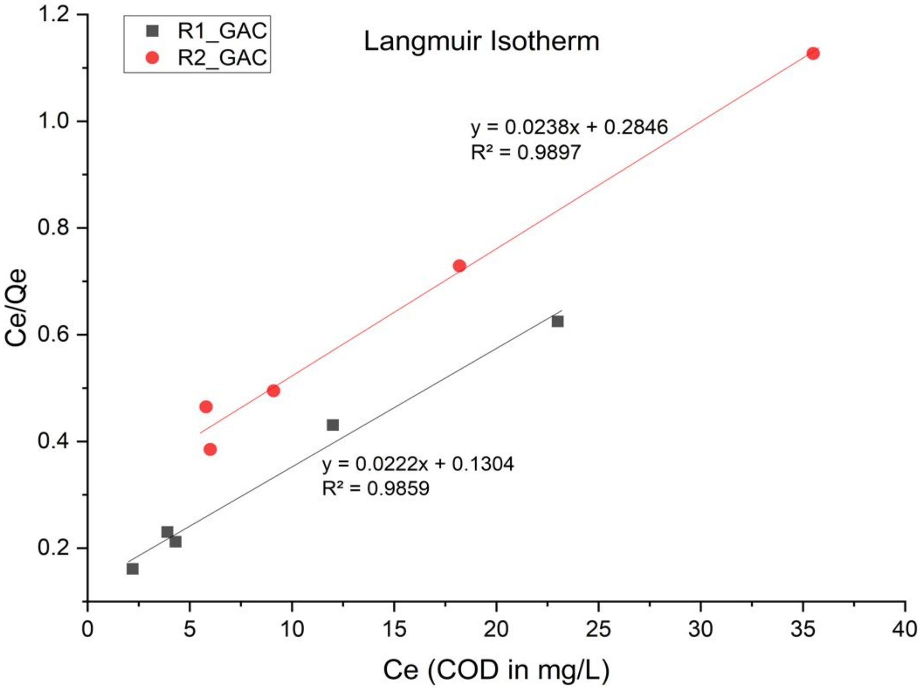

Figure S6**.** Langmuir isotherm model for GAC adsorption of COD from R1 and R2

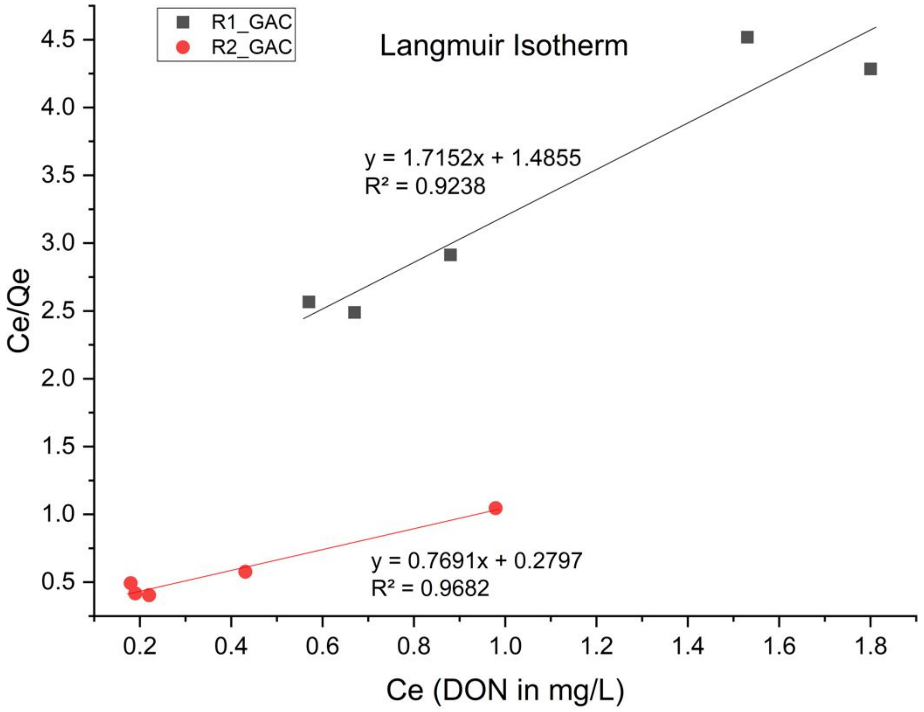

Figure S7. Langmuir isotherm model for GAC adsorption of DON from R1 and R2.

Table S6. Langmuir isotherm parameters for GAC adsorption of COD and DON from R1 and R2

Langmuir Model		Q_{max}	b	R^2	C_0 (mg/L)
**COD**	R1_GAC	45.04505	0.170245	0.9859	207.0
	R2_GAC	42.01681	0.083626	0.9897	193.0
**DON**	R1_GAC	0.583022	1.154628	0.9238	3.9
	R2_GAC	1.300221	2.749732	0.9682	5.6

To further analyze the adsorption characteristics, the Freundlich isotherm equation was employed, with the intercept and slope of the linear regression results used to calculate the Freundlich parameters K_F and 1/n. The findings are presented in Figures S8 and S9, with the Freundlich parameters summarized in Table S4. The correlation coefficients indicated a strong linear relationship for both COD and DON, with r^2 values ranging from 0.89 to 0.98. All models exhibited n values less than 1.00, suggesting relatively weak adsorption and a decrease in adsorption intensity with increasing concentration. The adsorption rate was highest for R1 DON and lowest for R1 COD, as demonstrated by the order of K_F values: R1 DON > R2 DON > R2 COD > R1 COD. Notably, COD displayed a greater maximum adsorption capacity than DON, consistent with its initial higher concentration.

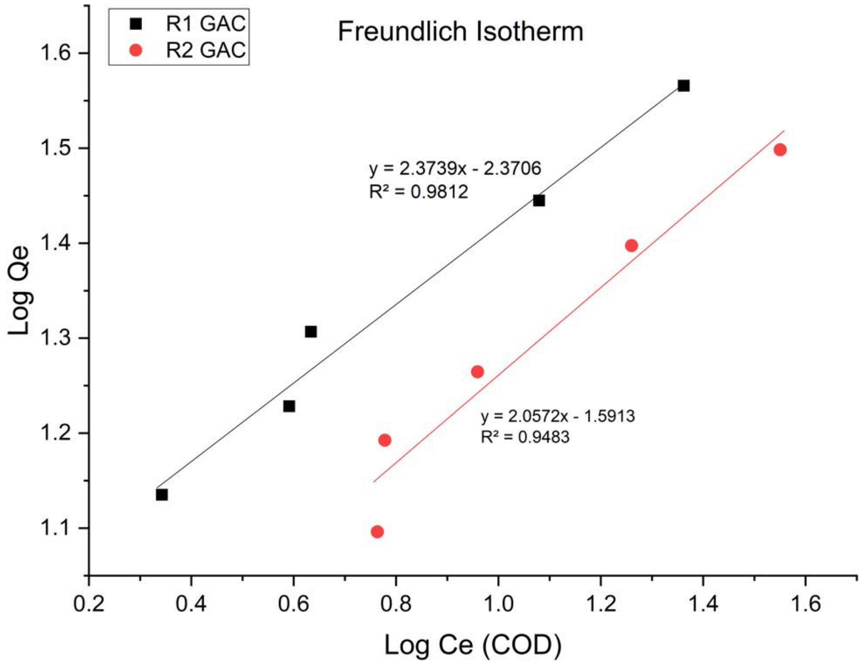

Figure S8. Freundlich isotherm model for GAC adsorption of COD from R1 and R2.

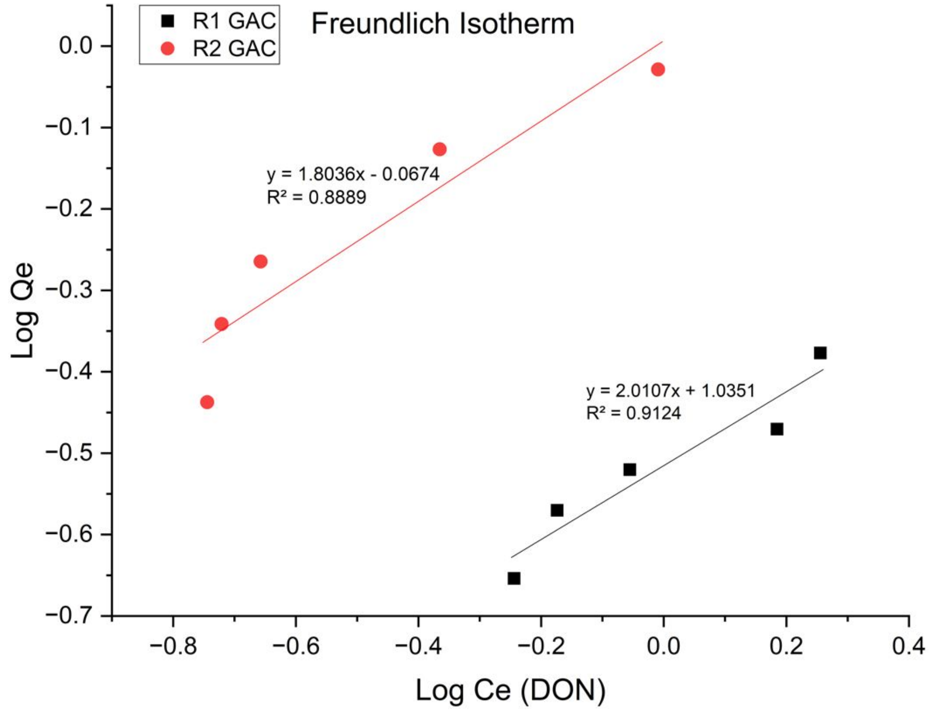

Figure S9. Freundlich isotherm model for GAC adsorption of DON from R1 and R2.

While the Langmuir parameters indicated comparable capacities for COD adsorption across the two reactors, there was a discrepancy in the adsorption capacity of DON, which was higher in R1 than in R2. The Freundlich equation fit poorly at low concentrations, indicating that the adsorption capacity values for DON may have been limited by its substantially lower initial concentration relative to COD [8]. Given the superior fit of the Langmuir model to the data compared to the Freundlich model and the unsuitability of the Freundlich model for low-concentration adsorption, the findings from the Freundlich model were rejected.

Table S7. Freundlich isotherm parameters for GAC adsorption of COD and DON from R1 and R2

**Freundlich Model**		**K_F**	**n**	**Q_M (mg/g)**	**R^2**	**C_0 (mg/L)**
**COD**	R1	0.0043	0.421	1340.5	0.981	207.0
	R2	0.0256	0.486	1289.9	0.948	193.0
**DON**	R1	10.839	0.497	167.3	0.912	3.9
	R2	0.856	0.554	18.9	0.889	5.6

4. Organic Matter characterization

4.1 Fourier-Transform Infrared (FTIR) Spectroscopy

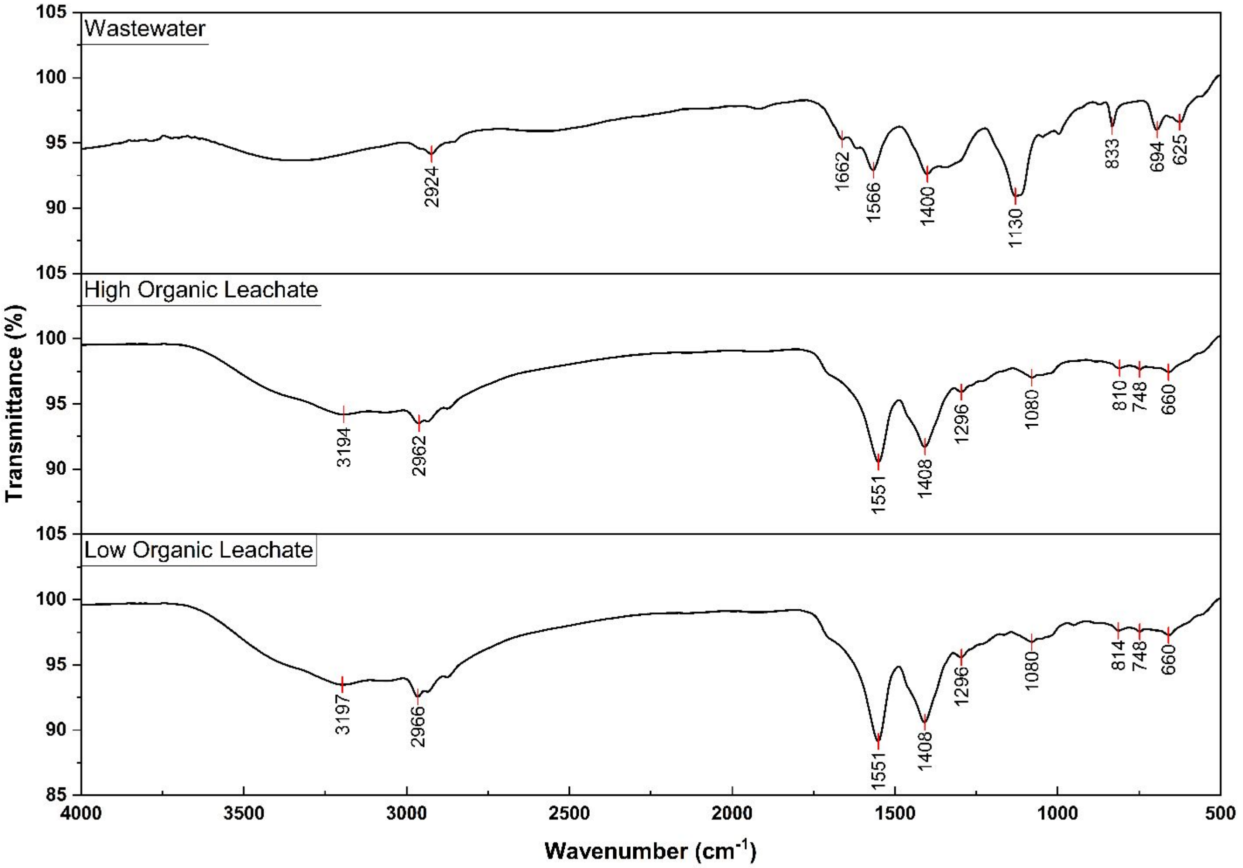

Figure S10. FTIR spectra for high organic and low organic leachate, and wastewater.

4.2 ^{13}C Nuclear Magnetic Resonance (^{13}C NMR) Spectroscopy

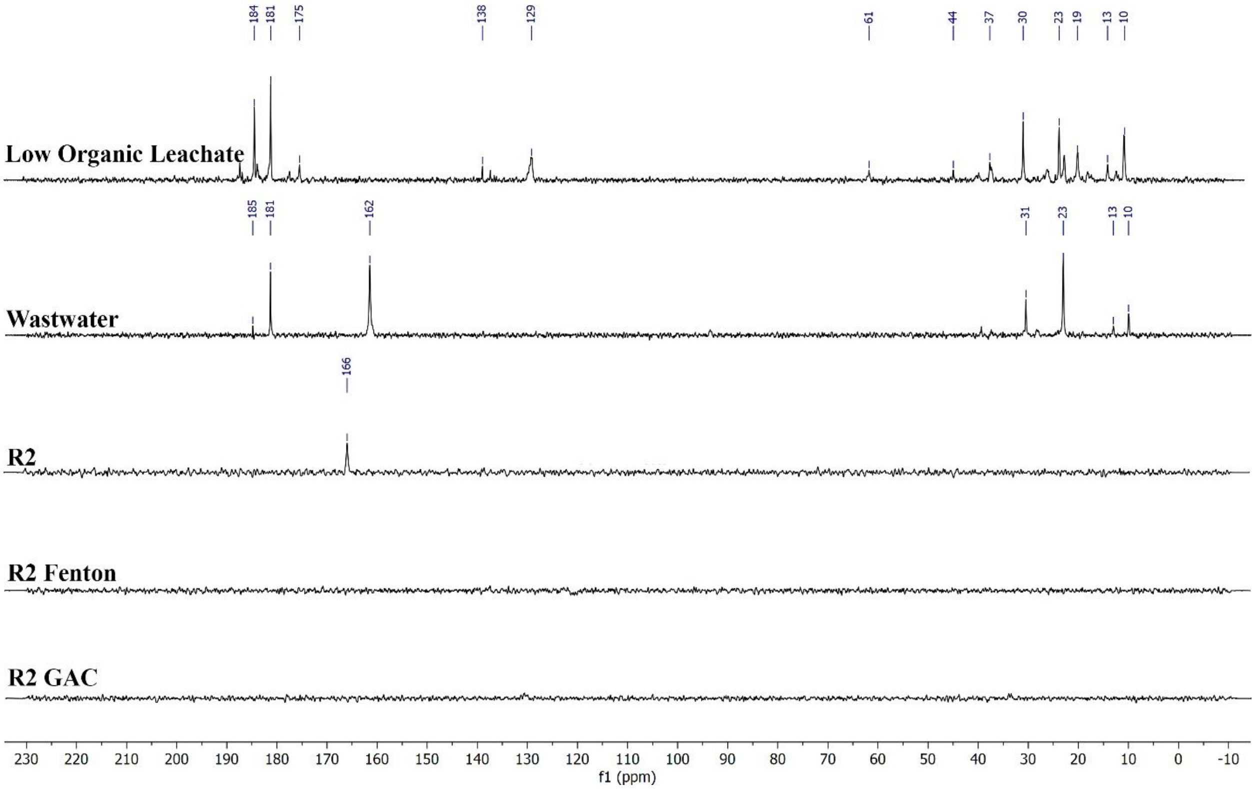

Figure S11: ^{13}C NMR spectra for low organic leachate, wastewater, and R2 effluent before and after the Fenton and GAC treatment.

5. Algal Bioassay

The Total chlorophyll B (chlorophytes) concentration followed a similar trend as Total chlorophyll A. In R1 treated with Fenton, concentrations increased from 0.43 µg/L on Day 1 to 0.83 µg/L on Day 4, then sharply decreased to 0.05 µg/L by Day 7, indicating that the algal community may have experienced nutrient depletion and/or toxicity. GAC treatment in R1 resulted in a decrease from 0.58 µg/L on Day 1 to 0.33 µg/L on Day 4, further declining to 0.10 µg/L by Day 7, reinforcing the notion that GAC limits algal growth. In R2, Fenton treatment yielded chlorophyll b concentrations starting at 0.42 µg/L on Day 1, peaking at 0.85 µg/L on Day 4, then dropping to 0.27 µg/L by Day 7. GAC treatment resulted in an initial concentration of 0.58 µg/L on day 1, declining to 0.25 µg/L on day 4, and stabilizing at 0.07 µg/L by Day 7. Analysis of fucoxanthin (diatoms) concentrations further supports these observations. In R1, Fenton treatment resulted in an increase from 1.09 µg/L on Day 1 to 6.37 µg/L by Day 4, followed by a decrease to 1.50 µg/L by Day 7, indicating an initial stimulation of algal biomass but subsequent decline due to potentially unfavorable conditions. GAC treatment for R1 showed lower fucoxanthin (diatoms) concentrations, starting at 0.87 µg/L on Day 1, peaking at 1.22 µg/L on Day 4, and decreasing to 0.68 µg/L by Day 7. For R2, Fenton treatment resulted in a significant increase in fucoxanthin (diatoms), peaking at 5.18 µg/L on day 4 before decreasing to

2.38 µg/L by day 7. GAC treatment of R2 produced a stable concentration of fucoxanthin (diatoms), peaking at 1.00 µg/L on Day 7. The graphical representation of these results is shown in Figure S12.

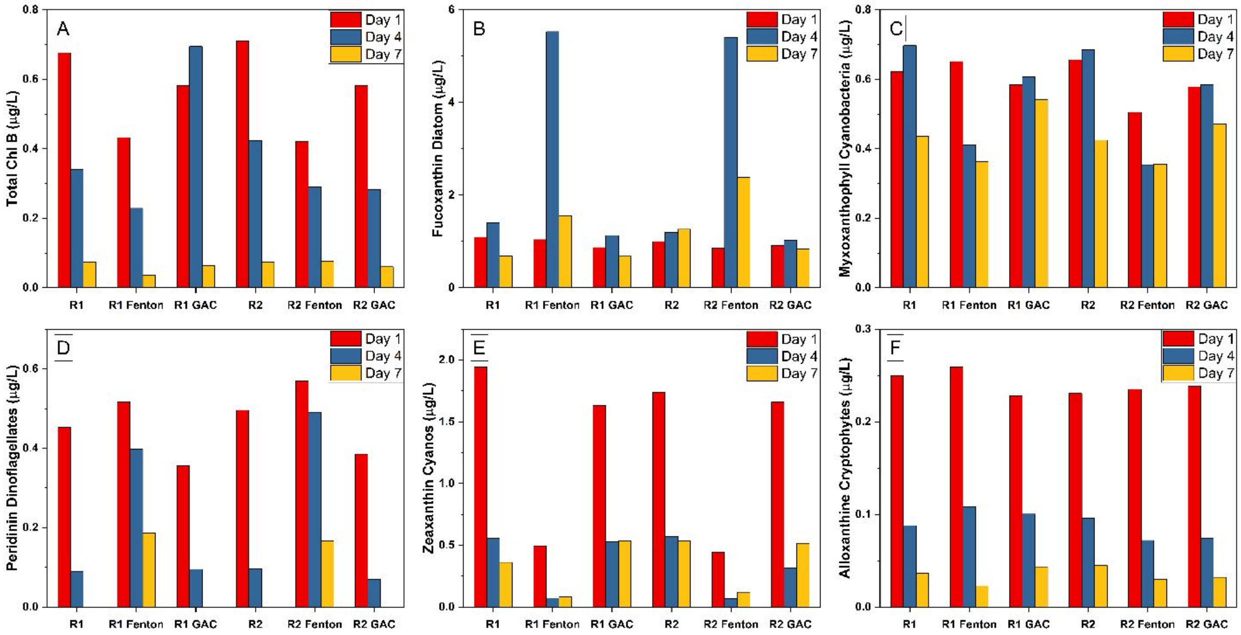

Figure S12: Temporal trend in different algal bioassay concentrations in R1 and R2 before and after the Fenton and GAC treatment.

Myxoxanthophyll (cyanobacteria) concentrations in R1 exhibited an initial increase from 0.62 µg/L on Day 1 to 0.67 µg/L on Day 4, followed by a decrease to 0.42 µg/L by Day 7 for Fenton treatment. GAC treatment led to a decline from 0.58 µg/L on Day 1 to 0.51 µg/L on Day 4 and further reduced to 0.28 µg/L by Day 7. In R2, myxoxanthophyll increased from 0.66 µg/L on Day 1 to 0.68 µg/L on Day 4 for Fenton, then dropped to 0.43 µg/L by Day 7, while GAC treatment resulted in a decrease from 0.58 µg/L on Day 1 to 0.52 µg/L on Day 4 and further down to 0.44 µg/L by Day 7. Peridinin (dinoflagellate) concentrations were notably influenced by the treatment. For R1 treated with Fenton, concentrations rose from 0.52 µg/L on Day 1 to 0.58 µg/L by Day 4 but then dropped to 0.15 µg/L by Day 7, indicating the potential adverse effects of residual contaminants. In GAC-treated R1, Peridinin (dinoflagellates) concentrations remained low, starting at 0.36 µg/L on Day 1 and decreasing to 0.10 µg/L by Day 7. For R2, Fenton treatment saw peridinin levels increase from 0.57 µg/L on Day 1 to 0.71 µg/L by Day 4 but declined to 0.15 µg/L by Day 7, suggesting a similar trend to R1. Zeaxanthin (cyanobacteria) concentrations in R1 showed an initial decline from 1.94 µg/L on Day 1 to 0.82 µg/L on Day 4 for Fenton treatment, stabilizing at 0.36 µg/L on Day 7. The GAC treatment resulted in a decrease from 1.63 µg/L on day 1 to 0.52 µg/L on Day 7. In R2, Zeaxanthin (Cyanobacteria) concentrations followed a similar pattern, decreasing from 1.74 µg/L on Day 1 to 0.98 µg/L on Day 4 for Fenton and stabilizing at 0.53 µg/L on Day 7. Finally, alloxanthin (cryptophyte) concentrations exhibited a steady decline over the observation period for both R1 and R2. In R1, Alloxanthin decreased from 0.25 µg/L on Day 1 to 0.05 µg/L by Day 7 for Fenton, while GAC

treatment showed a decrease from 0.23 µg/L to 0.03 µg/L. In R2, Alloxanthin concentrations fell from 0.23 µg/L on Day 1 to 0.02 µg/L on Day 7 for Fenton and from 0.24 µg/L to 0.02 µg/L for GAC.

6. Experimental set up

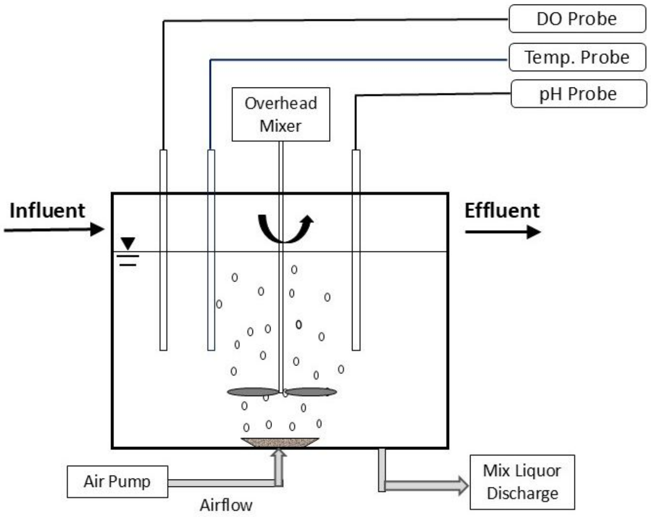

Figure S13: SBR setup for treating inorganic and dissolved organic nitrogen from a mixture of landfill leachate and wastewater.

7. Characteristics of GAC

Table S8. Characteristics of GAC

Parameter	Value
**BET Surface area (m^2/g)**	1517.88
**Particle Size (µm)**	30 - 60
**Mesopore Volume (cm^3/g)**	0.22
**Micropore Volume (cm^3/g)**	0.34
**pH_{pzc}**	7.1

References

(1) Bolyard, S. C.; Reinhart, D. R.; Richardson, D. Conventional and Fourier Transform Infrared Characterization of Waste and Leachate during Municipal Solid Waste Stabilization. *Chemosphere* **2019**, *227*, 34–42.

(2) Chen, N.; Huang, L. J.; Liu, N.; Liu, Y.; Wang, S. F. Analysis of the Effect of Erythromycin Wastewater Degradation by Fenton Method. *Asian J. Chem.* **2013**, *25* (13), 7208–7210.
(3) Cheng, Y.; Chen, Y.; Lu, J.; Nie, J.; Liu, Y. Fenton Treatment of Bio-Treated Fermentation-Based Pharmaceutical Wastewater: Removal and Conversion of Organic Pollutants as Well as Estimation of Operational Costs. *Environ. Sci. Pollut. Res.* **2018**, *25*, 12083–12095.
(4) Nousheen, R.; Batool, A.; Rehman, M. S. U.; Ghufran, M. A.; Hayat, M. T.; Mahmood, T. Fenton-Biological Coupled Biochemical Oxidation of Mixed Wastewater for Color and COD Reduction. *J. Taiwan Inst. Chem. Eng.* **2014**, *45* (4), 1661–1665.
(5) Sun, Y.; Hua, X.; Ge, R.; Guo, A.; Guo, Z.; Dong, D.; Sun, W. Investigation on Pretreatment of Centrifugal Mother Liquid Produced in the Production of Polyvinyl Chloride by Air-Fenton Technique. *Environ. Sci. Pollut. Res.* **2013**, *20*, 5797–5805.
(6) Babuponnusami, A.; Muthukumar, K. A Review on Fenton and Improvements to the Fenton Process for Wastewater Treatment. *J. Environ. Chem. Eng.* **2014**, *2* (1), 557–572.
(7) Martınez, N. S. S.; Fernández, J. F.; Segura, X. F.; Ferrer, A. S. Pre-Oxidation of an Extremely Polluted Industrial Wastewater by the Fenton's Reagent. *J. Hazard. Mater.* **2003**, *101* (3), 315–322.
(8) Rouquerol, J.; Rouquerol, F.; Llewellyn, P.; Maurin, G.; Sing, K. *Adsorption by Powders and Porous Solids: Principles, Methodology and Applications*; Academic press, 2013.
